# Identification of Human Proteins That Modify Misfolding and Proteotoxicity of Pathogenic Ataxin-1

**DOI:** 10.1371/journal.pgen.1002897

**Published:** 2012-08-16

**Authors:** Spyros Petrakis, Tamás Raskó, Jenny Russ, Ralf P. Friedrich, Martin Stroedicke, Sean-Patrick Riechers, Katja Muehlenberg, Angeli Möller, Anita Reinhardt, Arunachalam Vinayagam, Martin H. Schaefer, Michael Boutros, Hervé Tricoire, Miguel A. Andrade-Navarro, Erich E. Wanker

**Affiliations:** 1 Neuroproteomics, Max Delbrueck Center for Molecular Medicine, Berlin, Germany; 2 Unité BFA (EAC 7059), Université Paris Diderot-Paris7/CNRS, Paris, France; 3 Computational Biology and Data Mining, Max Delbrueck Center for Molecular Medicine, Berlin, Germany; 4 Division of Signaling and Functional Genomics, German Cancer Research Center, Heidelberg, Germany; University of Minnesota, United States of America

## Abstract

Proteins with long, pathogenic polyglutamine (polyQ) sequences have an enhanced propensity to spontaneously misfold and self-assemble into insoluble protein aggregates. Here, we have identified 21 human proteins that influence polyQ-induced ataxin-1 misfolding and proteotoxicity in cell model systems. By analyzing the protein sequences of these modifiers, we discovered a recurrent presence of coiled-coil (CC) domains in ataxin-1 toxicity enhancers, while such domains were not present in suppressors. This suggests that CC domains contribute to the aggregation- and toxicity-promoting effects of modifiers in mammalian cells. We found that the ataxin-1–interacting protein MED15, computationally predicted to possess an N-terminal CC domain, enhances spontaneous ataxin-1 aggregation in cell-based assays, while no such effect was observed with the truncated protein MED15ΔCC, lacking such a domain. Studies with recombinant proteins confirmed these results and demonstrated that the N-terminal CC domain of MED15 (MED15CC) *per se* is sufficient to promote spontaneous ataxin-1 aggregation *in vitro*. Moreover, we observed that a hybrid Pum1 protein harboring the MED15CC domain promotes ataxin-1 aggregation in cell model systems. In strong contrast, wild-type Pum1 lacking a CC domain did not stimulate ataxin-1 polymerization. These results suggest that proteins with CC domains are potent enhancers of polyQ-mediated protein misfolding and aggregation *in vitro* and *in vivo*.

## Author Summary

Spinocerebellar ataxias (SCAs) are a group of inherited neurodegenerative diseases with around 30 subtypes, which are characterized by a progressive loss of cerebellar neurons. Neuronal death has been linked to the aggregation of mutated disease-causing proteins, such as ataxin-1 (ATXN1). Pathogenic ATXN1 contains an elongated glutamine stretch, which triggers spontaneous misfolding and self-assembly of the protein into aggregates. Earlier studies in lower organisms have discovered many non-human proteins that alter aggregation and/or toxicity of mutant ATXN1. Here, we combine an experimental screening approach with bioinformatics to find human proteins that modulate aggregation and toxicity of ATXN1. We identified 21 proteins affecting mutant ATXN1 in mammalian cells. Further characterization revealed that enhancers of ATXN1-mediated toxicity contain α-helical coiled-coil domains as structural motifs, while suppressors do not. Detailed studies with the ATXN1 interacting proteins MED15 and Pum1 finally demonstrated that coiled-coil domains are indeed critical for the aggregation and toxicity promoting effects of human proteins. Our study contributes to a deeper understanding of ATXN1 aggregation and SCA1 pathogenesis and highlights potential therapeutic targets for further investigations.

## Introduction

Accumulation of misfolded proteins with pathogenic polyglutamine (polyQ) tracts in insoluble aggregates is a hallmark of late-onset neurodegenerative diseases, including Huntington's disease (HD) and spinocerebellar ataxias (SCAs) [1], [2]. Misfolded proteins in neurons are often deposited in cytoplasmic or nuclear inclusion bodies that can be readily detected with immunohistological methods in brains of patients and transgenic mice [3]. Experimental evidence was presented that aggregation of disease proteins in model organisms is associated with neuronal dysfunction and toxicity [4], suggesting that this process is causally linked to pathogenesis. However, data indicating that formation of inclusion bodies may be protective for mammalian cells have also been reported [3]. Insoluble protein aggregates are less mobile than soluble polyQ proteins and therefore have a diminished ability to associate with essential cellular factors [5], [6]. Thus, the question whether polyQ-mediated protein aggregation is deleterious or protective for cells still remains unanswered.

Various experimental studies indicate that pathogenic polyQ tracts with ∼40 or more glutamine residues drive spontaneous misfolding and aggregation of disease proteins such as huntingtin (HTT), ataxin-1 (ATXN1) and ataxin-3 (ATXN3) [7], [8]. The structural basis of polyQ-mediated protein aggregation is believed to be the formation of “polar zippers”, in which β-sheets are stabilized by hydrogen bonds between polar amino acids [9]. Once monomers of polyQ-proteins are joined together by hydrogen bonds they efficiently self-assemble into large, ordered protein aggregates with a characteristic fibrillar cross β-sheet structure [7], [8].

The kinetics of spontaneous polyQ-mediated protein assembly is influenced by the concentration, the amino acid composition and the size of a disease protein. For instance, full-length HTT, with a pathogenic polyQ tract (≥40 glutamines), does not spontaneously self-assemble into insoluble protein aggregates in mammalian cells [10]. In contrast, short N-terminal HTT fragments with pathogenic polyQ sequences form aggregates very efficiently in various disease model systems [11], [12]. Similar results were obtained with other polyQ proteins such as ATXN1 or ATXN3 [13], suggesting that proteolytic cleavage of disease proteins in patient brains is a prerequisite for polyQ-mediated protein aggregation. This hypothesis was confirmed recently in a study that demonstrated ATXN3 processing to be critical for spontaneous protein aggregation in iPSC-derived neurons from SCA3 patients [14]. Also, experimental evidence has been presented that specific domains in disease proteins influence their propensity to spontaneously form insoluble aggregates. In ATXN1, e.g., a C-terminal self-association region (SAR) crucial for spontaneous polyQ-mediated protein aggregation was identified [15]. Deleting this region reduced the ability of pathogenic ATXN1 to form inclusion bodies in disease model systems; however, proteotoxicity and neurodegeneration were still detectable [16], supporting the view that neurotoxicity is caused by soluble proteins with expanded polyQ tracts rather than by insoluble protein aggregates.

In the last decade a number of proteins have been reported to influence misfolding and aggregation of polyQ-containing disease proteins in model systems like yeast, worm or flies [17]–[26]. These modulators are functionally involved in various cellular processes, including RNA metabolism, protein synthesis, protein folding and protein degradation. However, the molecular mechanisms by which such modulators control the toxicity of polyQ-containing disease proteins in model organisms are not well understood. Also, it remains to be investigated whether their human homologues show similar effects in mammalian cells and are relevant for disease pathogenesis.

In this study, we searched for human proteins that influence toxicity and aggregation of pathogenic ATXN1 in cell model systems. Using literature information on lower model organisms we first predicted ∼200 potential human modifiers. These were then systematically tested in cell-based assays. Using this approach, we identified 21 human proteins, most of which significantly influenced both toxicity and aggregation of pathogenic ATXN1 in mammalian cells. In computational studies we predicted that many ATXN1 toxicity enhancers contain putative coiled-coil (CC) domains. Strikingly, no such domains were predicted for toxicity suppressors, suggesting that CC regions in modifiers are critical for their aggregation and toxicity promoting effects on polyQ disease proteins. We then investigated in more detail whether the ATXN1 interacting proteins MED15 and Pum1 directly influence polyQ-mediated ATXN1 aggregation in cell-free assays. MED15 contains a relatively large computationally predicted N-terminal CC domain, while such a domain was not predicted for Pum1. We found that MED15 but not Pum1 efficiently promotes spontaneous ATXN1 aggregation *in vitro*, suggesting that the N-terminal CC is critical for this aggregation enhancing effect. Further studies with recombinant proteins demonstrated that the N-terminal CC of MED15 (MED15CC) alone is sufficient to promote spontaneous ATXN1 aggregation in cell-free assays. We also observed that a hybrid Pum1-MED15CC fusion protein potently enhances ATXN1 aggregation in mammalian cells, while such an effect was not observed with the wild-type Pum1 protein. Finally, cell-based assays revealed that a truncated MED15 fragment lacking the N-terminal CC (MED15ΔCC) in contrast to the wild-type protein does not promote spontaneous ATXN1 aggregation. Together, this supports the hypothesis that CC domains in interacting proteins are critical for stimulation of spontaneous ATXN1 aggregation. The potential implications of these results for the pathogenesis of polyQ disorders and the development of novel therapeutic strategies are discussed.

## Results

### Selection of human polyQ toxicity and aggregation modulators

Previously, proteins that modify the toxicity and aggregation of different polyQ-containing disease proteins have been identified using various model organisms, including *Caenorhabditis elegans* [23], [24], *Drosophila melanogaster* [17]–[20], [22], [25] *Saccharomyces cerevisiae* [21], [26] and *Mus musculus* [27]. We selected 192 human homologues of known modifiers identified in lower organisms according to information in the HomoloGene database [28]. In addition, 45 human proteins that associate with polyQ disease proteins in yeast two-hybrid (Y2H) and co-immunoprecipitation assays were chosen [29], because modulators of polyQ toxicity and aggregation are reportedly enriched among direct interaction partners of disease proteins [30]. In total, 237 genes encoding potential human modulator proteins were defined, 202 of which (87%) were available as full-length cDNAs for modifier screens in a cell model system (Table S1). The selected cDNAs were cloned into the mammalian expression vector pCMV-FLAG using the GATEWAY technology [31]; the identity of genes was confirmed by DNA restriction and sequencing.

For expression analysis, 25 plasmids were randomly selected and transfected into COS-1 cells. Synthesis of N-terminally FLAG-tagged recombinant fusion proteins was verified by SDS-PAGE and immunoblotting. We verified 22 of 25 randomly selected plasmids (88%) to express FLAG-tagged proteins at approximately the expected size in mammalian cells (Figure S1), suggesting that most constructs were suitable for modulator screening in cell-based assays.

### Identifying modulators of YFP-ATXN1Q82^NT^ toxicity and aggregation

**Figure 1:**
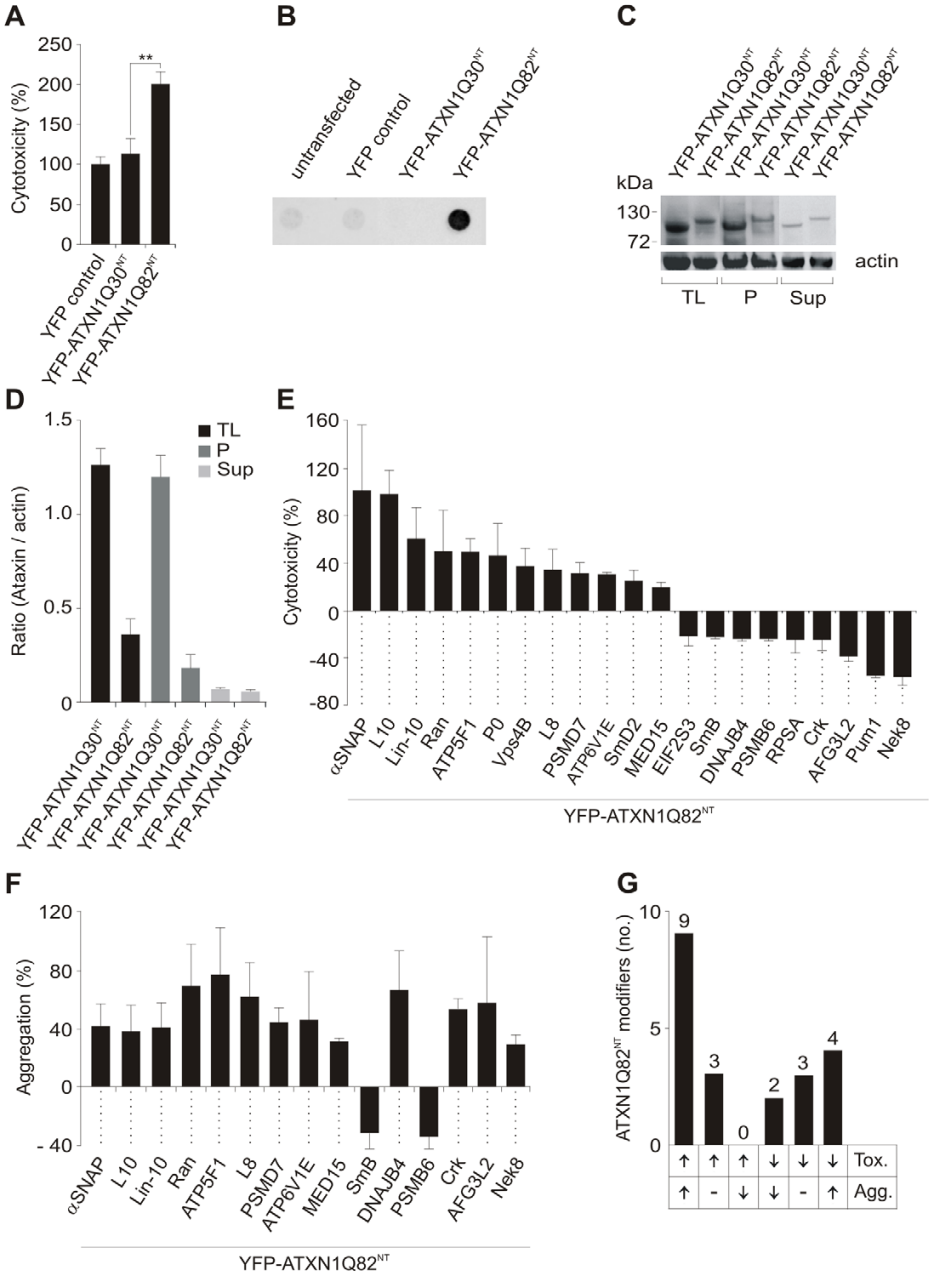
Identification of ATXN1 toxicity and aggregation modifiers using cell-based assays. (A) Relative caspase 3/7 activity change induced by overproduction of proteins YFP-ATXN1Q30^NT^ or YFP-ATXN1Q82^NT^ in COS-1 cells compared to YFP overproducing cells. Caspase 3/7 activity was significantly increased in cells producing pathogenic YFP-ATXN1Q82^NT^ compared to non-pathogenic YFP-ATXN1Q30^NT^ (Student's t-test, ** p<0.01, n = 3). Error bars indicate SD. (B) Detection of SDS insoluble protein aggregates by filter retardation assay. The protein YFP-ATXN1Q82^NT^ but not YFP-ATXN1Q30^NT^ forms SDS-stable protein aggregates in COS-1 cells. Equal amounts of total protein were loaded. FLAG control protein was set to 100%. (C) Western blot detection of YFP-ATXN1Q30^NT^ and YFP-ATXN1Q82^NT^ fusion proteins in total COS-1 cell extracts, supernatant and pellet samples. The pellet samples were dissolved in 8 M urea. Proteins were detected using an anti-GFP monoclonal antibody (Sigma). Actin protein levels were used as a loading control; Abbreviations: TL - total lysate; P – pellet; Sup – supernatant. (D) Quantification of YFP-tagged ATXN1 fusion proteins using the AIDA densitometry software. Abbreviations: TL - total lysate; P – pellet; Sup – supernatant. (E) Effects of modifier proteins on YFP-ATXN1Q82^NT^-induced cellular toxicity. 12 proteins were defined as enhancers and 9 as suppressors of YFP-ATXN1Q82^NT^ toxicity. (F) Effects of modifier proteins on YFP-ATXN1Q82^NT^ aggregation monitored by filter retardation assays. 15 of 21 YFP-ATXN1Q82^NT^ toxicity modulators also influence polyQ-mediated protein aggregation. Data is shown as mean ± SD from three independent experiments, which were performed in triplicates. Student's t-tests were used for statistical comparisons, p<0.05. (G) A comparison of modifier effects on YFP-ATXN1Q82^NT^ cytotoxicity and aggregation.

First, a cell-based assay for the detection of human proteins that influence the toxicity of an N-terminal, aggregation-prone mutant ATXN1 fragment was established (Figure S2A). Plasmids encoding YFP-tagged truncated ATXN1 fusion proteins with non-pathogenic or pathogenic polyQ tracts (YFP-ATXN1Q30^NT^ and ATXN1Q82^NT^) were constructed and transiently transfected into COS-1 cells. After 48 h toxicity was monitored using a standard caspase-3/7 assay. We observed that caspase-3/7 activity was ∼2-fold higher in cells with pathogenic YFP-ATXN1Q82^NT^ than in cells with non-pathogenic YFP-ATXN1Q30^NT^ or the control protein YFP (Figure 1A), indicating that the toxicity of ATXN1 fusion proteins in COS-1 cells is polyQ length dependent.

Next, we examined whether the proteins YFP-ATXN1Q30^NT^ and YFP-ATXN1Q82^NT^ spontaneously form SDS-stable protein aggregates in COS-1 cells using a filter retardation assay [32]. This method allows the quantification of large SDS-stable protein aggregates, whereas soluble monomers and small oligomers of polyQ disease proteins are not detectable. We found that COS-1 cells overproducing YFP-ATXN1Q82^NT^ contain insoluble protein aggregates after 48 h, while such structures were not observed in cells overproducing YFP-ATX1Q30^NT^ (Figure 1B). This supports previously published studies that ATXN1 fragments with pathogenic polyQ tracts form SDS-stable protein aggregates in mammalian cells [16].

The formation of insoluble YFP-tagged ATXN1Q30^NT^ and YFP-ATXN1Q82^NT^ aggregates was also examined by confocal microscopy and quantified with a high content fluorescence imaging system (Figure S2B and S2C), showing both proteins to form similar numbers of cytoplasmic inclusion bodies in COS-1 cells. Thus, both YFP-ATXN1Q30^NT^ and YFP-ATXN1Q82^NT^ seem to be aggregation-prone proteins. However, SDS-stable protein aggregates, detectable by filter retardation assays, are only observed with pathogenic YFP-ATXN1Q82^NT^ (Figure 1B).

Finally, we investigated whether the expression levels of YFP-tagged ATXN1 fusion proteins with pathogenic and non-pathogenic polyQ tracts are similar in COS-1 cells. Soluble (supernatant) and insoluble protein fractions (pellet) were prepared from crude COS-1 cell extracts by centrifugation and analyzed by SDS-PAGE and immunoblotting (Figure 1C and 1D). We found that the non-pathogenic protein YFP-ATXN1Q30^NT^ is produced at higher amounts in COS-1 cells than the pathogenic protein YFP-ATXN1Q82^NT^, indicating that the observed increase in caspase-3/7 activity in YFP-ATXN1Q82^NT^ overproducing cells (Figure 1A) is not caused by a simple dosage effect. These investigations also confirmed the results obtained by immunofluorescence microscopy (Figure S2B and S2C), indicating that both YFP-ATXN1Q30^NT^ and YFP-ATXN1Q82^NT^ form similar amounts of insoluble protein aggregates in COS-1 cells.

The screening strategy for YFP-ATXN1Q82^NT^ toxicity modifiers in cell-based assays is schematically shown in Figure S3. COS-1 cells were co-transfected with pairs of plasmids encoding toxic YFP-ATXN1Q82^NT^ and one of the 202 selected modulator proteins. After 48 h caspase-3/7 activity was quantified in cell extracts. Modulators that enhanced or suppressed YFP-ATXN1Q82^NT^ toxicity compared to the control plasmid pFLAG-GW in three independent experiments by at least 20% were selected. Hits from primary screens were systematically re-tested in a control toxicity assay, monitoring the effect of modulator proteins on caspase-3/7 activity in the absence of YFP-ATXN1Q82^NT^ (Figure S3). Using this approach, 21 human proteins were identified that only influence caspase-3/7 activity in mammalian cells in the presence of YFP-ATXN1Q82^NT^ (Figure 1E and Table S2). Of these, 19 are human homologues of known modifiers of polyQ toxicity or aggregation in lower model organisms [19], [20], [24]. Two proteins (Crk and Lin-10) were previously identified as ATXN1 interaction partners in yeast two-hybrid screens [29]. We found that over-production of 12 of these proteins significantly enhanced YFP-ATXN1Q82^NT^-mediated toxicity in COS-1 cells, while 9 proteins were suppressors of toxicity. Overproduction of all identified 21 modifier proteins in COS-1 cells was confirmed by SDS-PAGE and immunoblotting (Figure S4).

Next, a membrane filter retardation assay [32] was utilized to examine the effects of toxicity modulators on YFP-ATXN1Q82^NT^ aggregation. COS-1 cells were co-transfected with pairs of plasmids encoding the modulators and YFP-ATXN1Q82^NT^. Formation of insoluble aggregates was quantified after 48 h by filtration of cell extracts through a cellulose acetate membrane. We found that 15 (71%) of 21 tested proteins also significantly influence YFP-ATXN1Q82^NT^ aggregation in cell-based assays (Figure 1F and Table S2), suggesting that the phenomena of toxicity and aggregation are causally linked. Production of 13 proteins enhanced YFP-ATXN1Q82^NT^ aggregation in COS-1 cells, while two proteins acted as aggregation suppressors.

We also systematically compared the results from toxicity and aggregation assays to identify proteins with different combined effects (Figure 1G). We found that 9 of the 21 tested modulator proteins (43%) enhance both YFP-ATXN1Q82^NT^ toxicity and aggregation in COS-1 cells. Three proteins were identified that enhance YFP-ATXN1Q82^NT^ toxicity, but do not significantly affect polyQ-mediated protein aggregation (Figure 1G). Overproduction of two proteins – the proteasome subunit beta type-6 (PSMB6) and the small nuclear ribonucleoprotein polypeptide B (SmB) - decreased both YFP-ATXN1Q82^NT^ toxicity and aggregation in cell assays, while three proteins were identified that reduced toxicity but did not significantly affect polyQ-mediated protein aggregation. Finally, four proteins (DNAJB4, AFG3L2, Nek8 and Crk) caused a reduction of YFP-ATXN1Q82^NT^ toxicity but at the same time increased YFP-ATXN1Q82^NT^ aggregation (Figure 1E–1G), suggesting that the deposition of polyQ-containing proteins is beneficial for mammalian cells under certain cellular conditions.

Finally, we also examined whether the identified toxicity modulators are specific for the YFP-ATXN1Q82^NT^ fusion protein. To address this question a cell-based toxicity and aggregation assay for the unrelated polyQ disease protein ATXN3 (YFP-ATXN3Q73) was established (Figure S5A and S5B) and applied for systematic modifier testing. COS-1 cells were co-transfected with pairs of plasmids encoding the toxic YFP-ATXN3Q73 fusion protein and one of the 21 selected modulator proteins; after 48 h caspase-3/7 activity in cell extracts was quantified. Strikingly, we found that only four (P0, Vps4B, Ran, Nek8) of the 21 previously identified YFP-ATXN1Q82^NT^ toxicity modulators (19%) influenced YFP-ATXN3Q73 toxicity in cell-based assays (Figure S5C and Table S2), indicating that most proteins are specific for pathogenic ATXN1. Two of the tested modulators (Vps4B and Ran) also enhanced YFP-ATXN3Q73 protein aggregation as shown in Figure S5D.

### RNAi knockdown studies in cell-based assays and transgenic flies confirm the effects of modulator proteins on ATXN1-mediated toxicity

**Figure 2:**
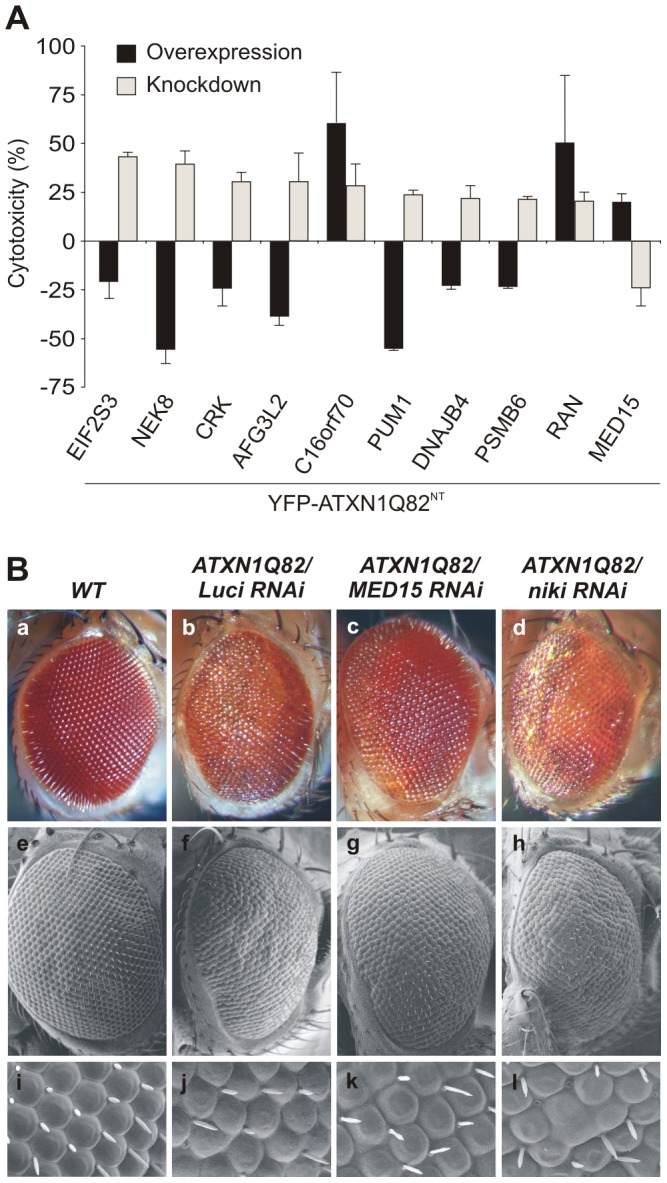
Reduced levels of modifier proteins influence the cytotoxicity of pathogenic ATXN1 in model systems. (A) Comparison of modifier gene effects on YFP-ATXN1Q82^NT^-induced toxicity in cell-based cDNA overexpression and siRNA knock-down experiments. Most modifiers in cDNA overexpression studies show opposing effects on YFP-ATXN1Q82^NT^ toxicity than modifiers in siRNA knock-down experiments. Error bars indicate SD from three independent experiments performed in triplicates. Student's t-tests were used for statistical comparisons, p<0.05. (B) Reduced levels of modifier proteins influence the toxicity of pathogenic ATXN1 in transgenic flies. RNAi knockdown of MED15 reduces ATXN1Q82-induced retinal degeneration, while knockdown of niki (homologue of human NEK8) increases the toxicity of the polyQ disease protein. The effects of modifiers on *Drosophila* eyes are visualized by loss of pigmentation (upper panel, optical microscopy), alterations in morphology of ommatidia (middle panel, electron microscopy) and bristle disorganization (lower panel, 10× magnifications of electron microscopy images). *Drosophila* eyes are shown from representative animals: a) wild-type (+/+), b) *GMR*-GAL4;UAS-ATXN1Q82/+;UAS-Luciferase RNAi, c) *GMR*-GAL4;UAS-ATXN1Q82/+; UAS-MED15 RNAi, and d) *GMR*-GAL4;UAS-ATXN1Q82/+;UAS-niki RNAi.

To investigate whether reduced levels of endogenous modulator proteins influence YFP-ATXN1Q82^NT^-induced toxicity, siRNA gene silencing experiments were performed in COS1 cells. In these experiments, we focused exclusively on the target genes identified in earlier cell-based overexpression studies (Figure 1E). Mammalian cells overproducing YFP-ATXN1Q82^NT^ were treated with siRNAs and caspase-3/7 activity in protein extracts was quantified after 48 h. siRNAs that increased caspase-3/7 activity in the absence of YFP-ATXN1Q82^NT^ in control experiments were not considered for further knock-down experiments (data not shown). We systematically tested 15 selected siRNAs in cell-based assays, 10 of which (67%) reproducibly influenced the toxicity of YFP-ATXN1Q82^NT^ (Figure 2A and Table S3). 9 siRNAs increased cellular toxicity, while one molecule decreased it. The knock-down of target gene expression was confirmed by qRT-PCR (Figure S6), confirming that upon siRNA treatment mRNA levels were reduced by ∼50–90%. A comparison of the results from siRNA knock-down and cDNA overexpression revealed that in eight of ten cases, knock-down of target genes had the opposite effect to gene overexpression on YFP-ATXN1Q82^NT^ toxicity (Figure 2A). However, we also found that both siRNA knock-down and overexpression of genes (C16orf70 and RAN) could promote the toxicity of YFP-ATXN1Q82^NT^ in cells.

Finally, we investigated whether knock-down of target genes influences the toxicity of full-length, pathogenic ATXN1 (ATXN1Q82) in the well-established SCA1 transgenic fly model [19]. We selected the proteins MED15 and Nek8 for these studies as they reproducibly influenced the toxicity of pathogenic YFP-ATXN1Q82^NT^ in several cell-based assays (Figure 1E and Figure 2A). We systematically crossed transgenic SCA1 flies with UAS-RNAi flies [33] and subsequently examined the degeneration of photoreceptors using scanning electron microscopy. We found that overproduction of ATXN1Q82 caused a pronounced neurodegenerative eye phenotype (Figure 2B), as previously reported [19]. However, ATXN1Q82-mediated eye degeneration was reduced in flies expressing the MED15 RNAi construct (Figure 2B), confirming the results from our cell model studies (Figure 2A). The opposite effect was observed in ATXN1Q82 flies that express a niki (Nek8 homologue) RNAi construct (Figure 2B), suggesting that expression of this gene protects against the toxicity in SCA1 transgenic flies. An eye phenotype was not detected when control wild-type flies with MED15 or niki RNAi constructs were analyzed (data not shown).

### Functional and structural properties of modulators influencing YFP-ATXN1Q82^NT^ toxicity

**Figure 3:**
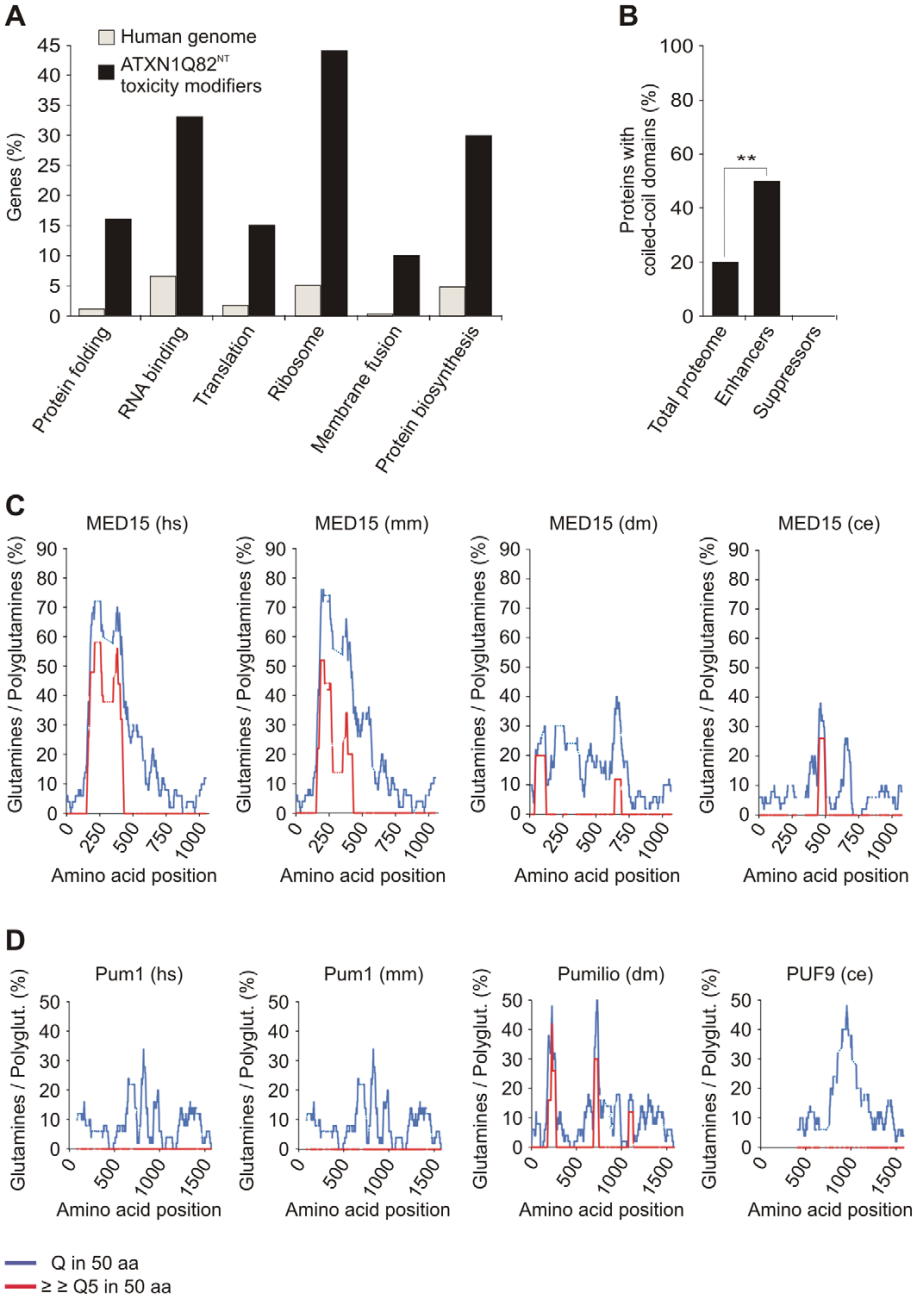
Specific functional categories and structural domains are over-represented among YFP-ATXN1Q82^NT^ toxicity modifiers. (A) Functional annotation of YFP-ATXN1Q82^NT^ toxicity modifiers was carried out using the EASE program. Genes encoding proteins involved in protein folding, RNA binding, translation, ribosome function, membrane fusion and protein biosynthesis are over-represented among YFP-ATXN1Q82^NT^ toxicity modifiers compared to the human genome (Fisher's exact test, p<0.05). (B) Proteins with coiled-coil domains are significantly enriched among YFP-ATXN1Q82^NT^ toxicity enhancers but not among suppressors (p = 0.0093; Chi-square test). Coiled-coil domains in modifier proteins were predicted using the COILS program (probability 0.8–1). Numbers of coiled-coil domain containing proteins were compared to the human proteome (73,427 proteins, Swiss-Prot database). (C) The human MED15 protein contains a conserved N-terminal Q-rich region with short polyQ tracts. The graphs represent the amounts of glutamines (Qs) in a window of 50 amino acids (blue line) or the amount of consecutive polyQ sequences (>5 Qs) in a window of 50 amino acids (red line). Abbreviations: hs – *Homo sapiens*, mm – *Mus musculus*, dm – *Drosophila melanogaster*, ce – *Caenorhabditis elegans*. (D) The human Pum1 protein contains conserved Q-rich regions but does not contain short polyQ tracts. For species abbreviations see C.

First, we investigated the potential functional roles of the 21 identified YFP-ATXN1Q82^NT^ toxicity modulators using the EASE program [34]. This tool identifies over-represented functional categories in gene lists. We observed that proteins involved in protein folding, RNA binding, translation, ribosome function, membrane fusion and protein biosynthesis are significantly enriched in the set of YFP-ATXN1Q82^NT^ toxicity modulators (adjusted p-value<0.05; Figure 3A). A detailed examination of the available literature information using PubMed data largely confirmed the results from the EASE analysis (Table S4). Among the most relevant functions of the 21 toxicity modulators, we found ribosomal proteins (P0, L8, L10 and RPSA) [35], well known regulators of translation and/or transcription (EIF2G, Pum1 or MED15) [36]–[38], proteins involved in mRNA splicing (Sm-D2 and SmB), vesicle trafficking (Ran, Vps4B, αSNAP and ATP6V1E), protein degradation (PSMD7 and PSMB6), protein folding (DNAJB4 and AFG3L2) and ATP metabolism (ATP5F1) (Table S4), confirming earlier reports that these cellular processes influence the toxicity of polyQ disease proteins [18], [24].

Recent studies have postulated that coiled-coil (CC) domains are critical for the spontaneous aggregation and toxicity of polyQ disease proteins [39], [40]. We therefore investigated computationally whether the identified YFP-ATXN1Q82^NT^ toxicity modulators contain such aggregation-promoting domains. Using the tool COILS [41], we predicted that 6 out of 21 proteins (28.6%) most likely contain CC domains (Table S5). Strikingly, we also found that among 12 toxicity enhancers, 6 (50%) contain putative CC domains (ATP6V1E, MED15, αSNAP, ATP5F1, PSMD7 and Vps4B), while this domain was not detected in any of the toxicity suppressors (9 proteins). This suggests that CC domains contribute to the toxicity promoting effects of modifier proteins in mammalian cells. We also observed that, compared to the human proteome, proteins with CC domains are significantly enriched among enhancers of YFP-ATXN1Q82^NT^ toxicity (p = 0.0093, Chi-square test; Figure 3B), supporting the importance of this observation.

Previous studies have also demonstrated that proteins with short, non-pathogenic polyQ tracts bind to neurodegenerative disease proteins with long, pathogenic polyQ tracts and influence their aggregation propensity *in vitro* and *in vivo* [42], [43]. This suggests that proteins with longer regions of polar amino acids might function as modifiers of polyQ-mediated ATXN1 misfolding and toxicity. Among the 21 human toxicity modifiers, we identified only two proteins (MED15 and Pum1) with Q-rich regions by computational sequence analysis (Figure 3C and 3D). MED15 contains a single, N-terminal Q-rich region with multiple short polyQ (5–16 Qs) tracts, which are also well conserved in orthologous proteins of lower organisms. Multiple conserved Q-rich regions were also detected in the protein Pum1. However, in this protein no polyQ tracts were detected (Figure 3C and 3D), suggesting that these Q-rich domains might have different functions and biochemical properties than the one in MED15.

Finally, we also investigated whether the Q-rich proteins MED15 and Pum1 contain conserved CC domains. Interestingly, we predicted that MED15 contains a long N-terminal CC domain, which is conserved in higher organisms (Figure S7A). Such a domain, however, was not predicted for Pum1 (Figure S7B). Obviously, these Q-rich proteins, which show opposite effects on YFP-ATXN1Q82^NT^ toxicity in cell-based assays (Figure 1E and Figure 2A), are structured differently with different functional consequences.

### The proteins MED15 and Pum1 interact with full-length ATXN1 in cell-based assays

**Figure 4:**
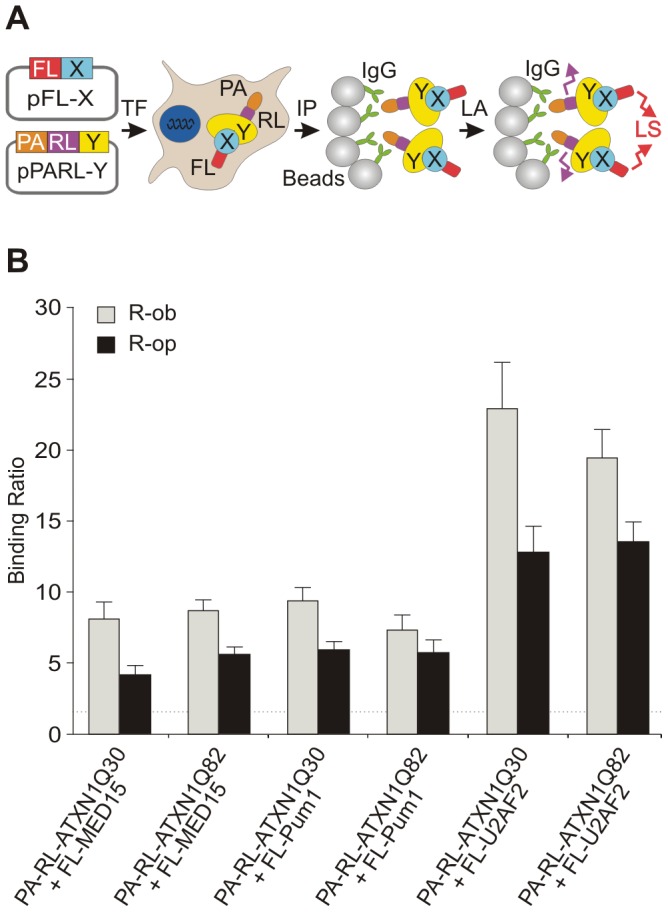
The modulator proteins MED15 and Pum1 interact with wild-type and mutant ATXN1. (A) Schematic representation of a LUMIER co-immunoprecipiation assay. The protein A (PA)-Renilla luciferase (RL)-tagged bait (PA-RL-Y) and the firefly luciferase (FL)-tagged prey proteins (FL-X, modifier) were co-produced in HEK293 cells. After co-immunoprecipitation from cell lysates the interaction between bait and prey fusion proteins was monitored by quantification of firefly luciferase activity in protein complexes. Quantification of Renilla luciferase activity in precipitated protein complexes indicates the immunoprecipitation of bait protein. Abbreviations: PA – protein A tag; RL – Renilla luciferase; FL – firefly luciferase; X – prey protein; Y – bait protein; TF – transfection of cells; IP –immunoprecipitation; LA – luminescence assay; LS – luminescence signal. (B) The human proteins U2AF2, MED15 and Pum1 interact with both wild-type and mutant ATXN1 fusion proteins in LUMIER co-immunoprecipitation assays. The R-op and R-ob binding ratios (see Figure S8) of >1.5 indicate that the proteins U2AF2, MED15 and Pum1 specifically interact with ATXN1Q30 or ATXN1Q82 in mammalian cells.

Previous investigations suggest that ATXN1 interacts with the human proteins Pum1 and MED15 [44]. To substantiate these observations, we examined whether the proteins associate with full-length wild-type or mutant ATXN1 (ATXN1Q30 or ATXN1Q82) using a LUminescence-based Mammalian IntERactome co-immunoprecipitation assay (LUMIER) [45]. The principle of this method is shown in Figure 4A and Figure S8. Full-length ATXN1 fusion proteins tagged with protein-A and Renilla luciferase (PA-RL-ATXN1Q30 and PA-RL-ATXN1Q82) were co-produced in HEK293 cells together with firefly luciferase (FL)-tagged Pum1 or MED15 (FL-Pum1 or FL-MED15). After 48 h protein complexes were co-immunoprecipitated with IgG-coated magnetic beads. Interactions were detected by quantification of firefly luciferase luminescence from co-immunoprecipitated protein complexes (Figure S8). We found that both Pum1 and MED15 specifically interact with both ATXN1Q30 and ATXN1Q82 in mammalian cells (Figure 4B; R-op and R-ob ratios >1.5), confirming previously published reports [44]. A similar result was obtained with the protein U2AF2 (positive control) [29]. Production of bait and prey proteins in mammalian cells was verified by the quantification of Renilla and firefly luciferase activities in cell extracts before the co-immunoprecipitation step (Figure S9). Thus, our studies support previous observations that both MED15 and Pum1 interact with wild type and mutant full-length ATXN1 proteins in mammalian cells.

### MED15 and Pum1 directly modulate polyQ-mediated ATXN1 aggregation in cell-free assays

**Figure 5:**
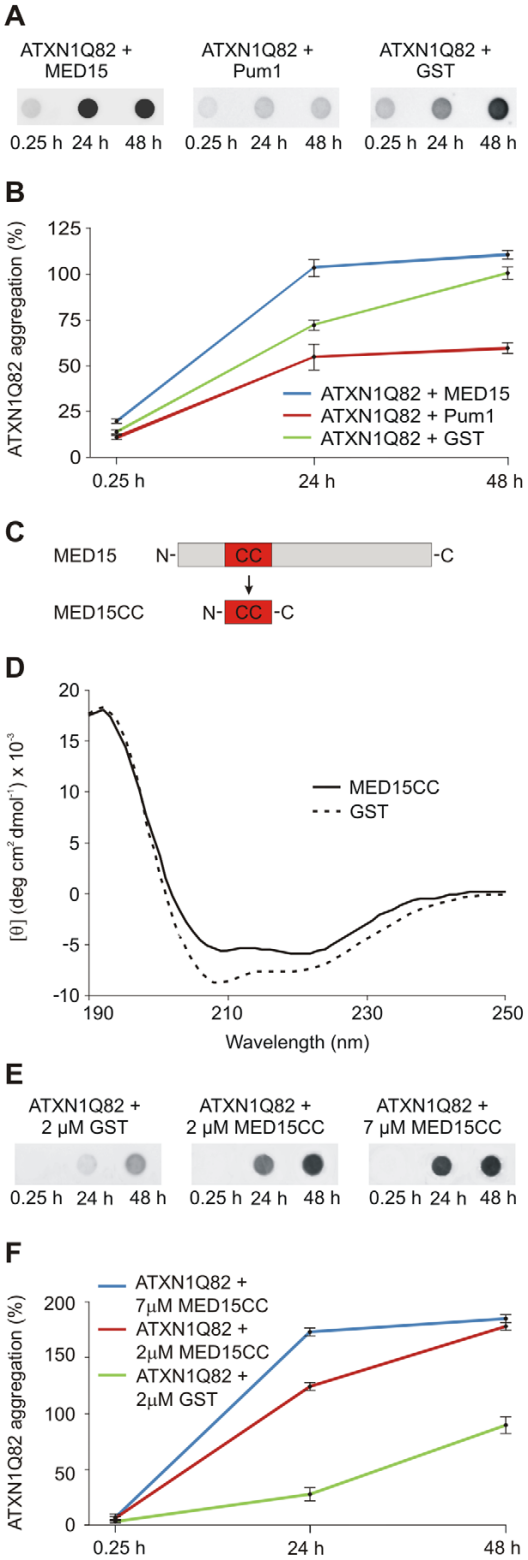
The N-terminal CC domain in MED15 enhances polyQ-mediated ATXN1 aggregation in cell-free assays. (A) Effects of His-tagged modifier proteins MED15 and Pum1 on spontaneous ATXN1Q82 aggregation *in vitro*. SDS-insoluble ATXN1Q82 aggregates were detected by filter assay. The protein MED15 enhanced spontaneous ATXN1Q82 aggregation while Pum1 showed the opposite effect. The protein GST was used as a control. Formation of SDS-insoluble ATXN1Q82 protein aggregates was detected by filter assay using the anti-ATXN1 antibody SA4645. (B) Quantification of ATXN1Q82 aggregates on filter membranes was performed using the AIDA densitometry software. The ATXN1Q82 immunoreactivity of samples obtained with the control protein GST was set to 100% (48 h). Error bars represent SD of three independent experiments. (C) Schematic view of the cloning strategy for the generation of the MED15CC fragment for *in vitro* aggregation experiments. A Gateway compatible entry plasmid encoding the N-terminal coiled-coil domain of MED15 (MED15CC) was constructed. (D) Far-UV CD spectra of the GST-tagged MED15CC fusion protein and GST. Both proteins adopt a typical alpha-helical conformation. A ratio of mean residue ellipticities ([θ]222/[θ]208) of 1.078948 indicates that MED15CC has a coiled-coil conformation. (E) The GST-tagged MED15CC fusion protein stimulates spontaneous ATXN1Q82 aggregation in cell-free assays. The formation of SDS-insoluble ATXN1Q82 aggregates was detected by filter retardation assays using the anti-ATXN1 antibody SA4645. (F) Quantification of insoluble ATXN1Q82 aggregates retained on filter membranes was performed using the AIDA densitometry software. The immunoreactivity of insoluble ATXN1Q82 aggregates in control samples with GST (48 h) was set to 100%. Error bars represent SD of three independent experiments.

We next investigated whether MED15 and Pum1 directly influence polyQ-mediated ATXN1 aggregation in cell-free assays. The principle of the *in vitro* ATXN1Q82 aggregation assay with purified proteins is schematically shown in Figure S10A. Recombinant proteins were produced as GST- and His-tagged fusions (GST-ATXN1Q82, His-MED15 and His-Pum1) in *E. coli* and purified to ∼90% homogeneity by affinity chromatography (Figure S10B). GST-ATXN1Q82 fusion protein was incubated with PreScission (PP) protease and the modifier proteins His-MED15 or His-Pum1; the formation of SDS-stable ATXN1Q82 aggregates was quantified after 24 and 48 h using a filter retardation assay [46]. PP was added to the reactions to remove the GST tag and to initiate spontaneous ATXN1Q82 aggregation [47]. We found that an equimolar concentration of His-MED15 stimulated *de novo* ATXN1Q82 aggregation *in vitro*, while His-Pum1 had the opposite effect (Figure 5A and 5B), indicating that both proteins directly influence spontaneous polyQ-mediated ATXN1 aggregation. An aggregation promoting or inhibiting effect was also observed when different concentrations of MED15 and Pum1 were added to reactions, respectively, indicating that the proteins influence ATXN1Q82 aggregation in a concentration-dependent manner (Figure S11A and S11B).

### A short N-terminal coiled-coil domain of MED15 is sufficient to promote ATXN1Q82 aggregation *in vitro*

Our data with cell-free and cell-based aggregation assays suggest that the conserved N-terminal CC domain in MED15 (Figure S7A) is critical for its aggregation-promoting effect on ATXN1. To investigate this possibility, a GST-tagged MED15CC fragment (aa 141–316; GST-MED15CC, Figure 5C) was produced in *E. coli*, purified by affinity chromatography and examined by circular dichroism (CD) spectroscopy [48]. The far-UV spectrum for MED15CC was acquired by subtraction of the CD spectrum of the control protein GST from that of the fusion protein GST-MED15CC (Figure 5D). The putative conformation of MED15CC was determined using the K2D2 secondary structure program [49]. We predicted that MED15CC is mainly an alpha-helical (55%) protein with a small percentage of beta-sheets (7%). Previous studies revealed that ratios of mean residue ellipticities at wavelengths 222 and 208 nm ([θ]222/[θ]208) above 1.0 indicate coiled-coil conformation [50], [51]. We determined that MED15CC has a [θ]222/[θ]208 ratio of 1.078, indicating that it forms a coiled-coil structure *in vitro*. For comparison, we observed a [θ]222/[θ]208 ratio of 0.84 for the control protein GST whose alpha-helical structure is known to lack coiled coils [52]. Thus, our structural investigations suggest that MED15CC has a characteristic alpha-helical CC structure.

Finally, we investigated whether MED15CC directly influences spontaneous ATXN1Q82 aggregation in cell-free assays. GST-ATXN1Q82 fusion protein (2 µM) was incubated with PP and GST-MED15CC (2 or 7 µM); the formation of SDS-stable ATXN1Q82 aggregates was quantified after 24 and 48 h by filter retardation assay (Figure 5E and Figure 5F). We found that the fusion protein GST-MED15CC in a concentration-dependent manner promoted spontaneous ATXN1Q82 aggregation *in vitro*.

### A hybrid Pum1-MED15CC protein promotes ATXN1 aggregation in cell-based assays

**Figure 6:**
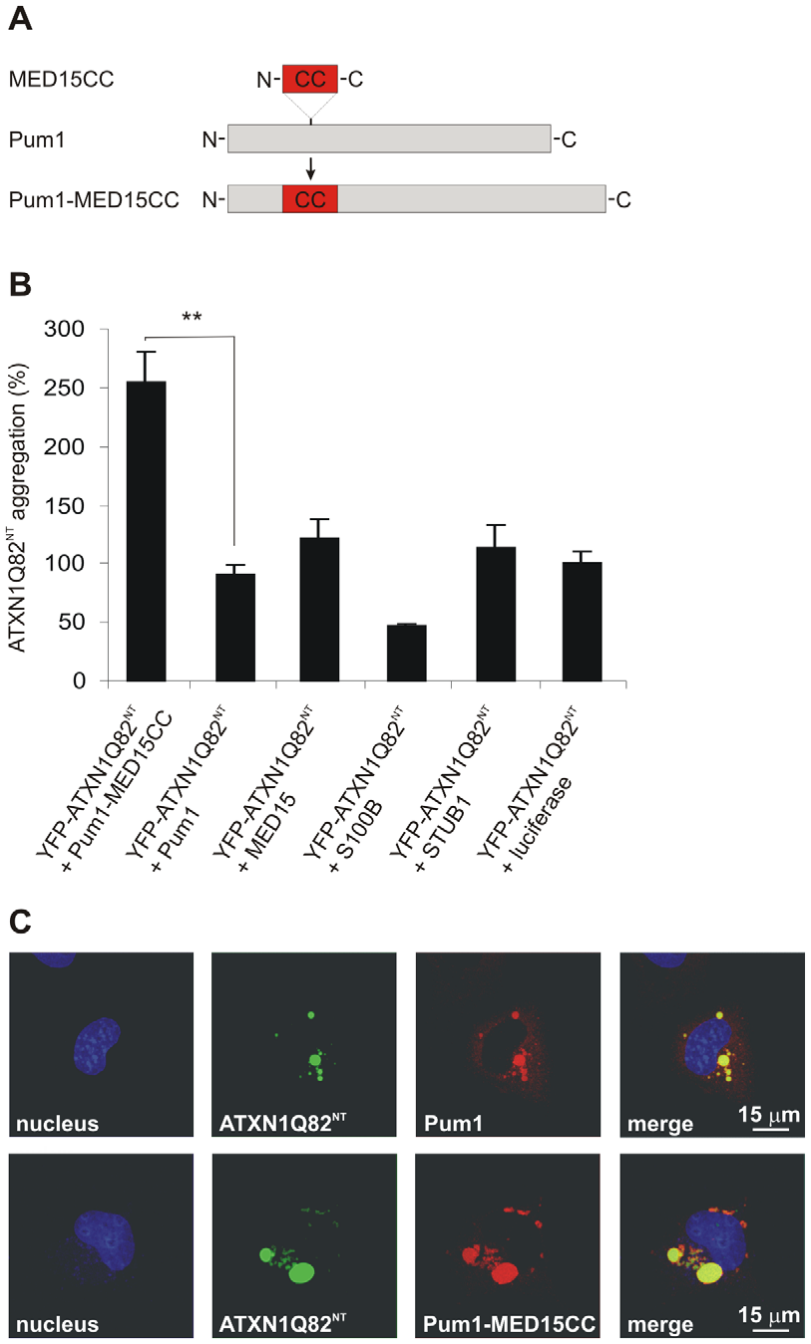
A hybrid Pum1 fusion protein with a MED15 CC domain is a potent enhancer of mutant ATXN1 aggregation in cell-based assays. (A) Schematic representation of the hybrid Pum1-MED15CC fusion protein. (B) Effects of mCherry-tagged proteins Pum1-MED15CC, Pum1, MED15, S100B, STUB1 and luciferase on spontaneous YFP-ATXN1Q82^NT^ aggregation in human neuroblastoma SH-EP cells. The formation of polyQ-containing ATXN1 aggregates was quantified by fluorescence imaging after 48 h. Data is normalized to the total mCherry fluorescence intensity. The proteins MED15, STUB1 and Pum1-MED15CC enhanced YFP-ATXN1Q82^NT^ aggregation compared to Pum1 or the luciferase control protein (100%). A suppression of YFP-ATXN1Q82^NT^ aggregation was observed with the control protein S100B. Data is shown as mean ± SD from three independent experiments. Experiments were performed in triplicates. Student's t-test was used for statistical comparisons, p<0.05. (C) Confocal microscopy images of COS-1 cells co-transfected with pairs of plasmids encoding YFP-ATXN1Q82^NT^ and mCherry-tagged Pum1 or Pum1-MED15CC. Nuclei were stained with Hoechst 33342 (blue). YFP-ATXN1Q82^NT^ aggregates were stained in green and mCherry-Pum1 or mCherry-Pum1-MED15CC in red color. Both Pum1 and Pum1-MED15CC proteins co-localize with YFP-ATXN1Q82^NT^ proteins.

Our data indicate that MED15CC alone is sufficient to promote spontaneous ATXN1Q82 aggregation in cell-free assays (Figure 5E and 5F). This suggests that an insertion of this domain into Pum1 (an ATXN1Q82 aggregation suppressor, Figure 5A) should increase its ability to promote ATXN1 aggregation. To test this hypothesis, we generated a plasmid for the production of a mCherry-tagged Pum1-MED15CC hybrid protein (Figure 6A). The MED15CC fragment was inserted in Pum1 in a putative unstructured region at amino acid 292, to avoid disrupting a folded protein domain. We also generated plasmids for the production of the mCherry-tagged proteins MED15, Pum1, S100B, STUB1 and luciferase, which were used as controls.

First, the effects of the recombinant proteins on spontaneous YFP-ATXN1Q82^NT^ aggregation were investigated (Figure 6B). The mCherry-tagged proteins were co-produced with YFP-ATXN1Q82^NT^ in a neuroblastoma cell model and formation of insoluble polyQ-containing protein aggregates was quantified after 48 h by high content fluorescence imaging [53]. We found that co-production of Pum1-MED15CC promoted YFP-ATXN1Q82^NT^ aggregation at levels significantly higher than Pum1 or the control protein luciferase, supporting the hypothesis that the MED15 CC domain is critical for aggregation stimulation. An aggregation-promoting effect was also obtained with the proteins MED15 and STUB1, while S100B showed the opposite effect, supporting previously published studies [54].

Next, we investigated whether the protein Pum1-MED15CC can also promote the aggregation of a full-length ATXN1 protein with a pathogenic polyQ tract (YFP-ATXN1Q82). In contrast to YFP-ATXN1Q82^NT^, this protein forms nuclear protein aggregates in mammalian cells (Figure S12A–S12D). We found that Pum1-MED15CC enhanced spontaneous YFP-ATXN1Q82 aggregation, while Pum1 had no effect, confirming the results obtained with the truncated ATXN1 fragment (Figure 6B).

Finally, we also investigated the association of Pum1-MED15CC and Pum1 with YFP-ATXN1Q82^NT^ by confocal microscopy (Figure 6C). We found that both mCherry-tagged proteins co-localize with perinuclear, cytoplasmic YFP-ATXN1Q82^NT^ aggregates in COS-1 cells, confirming the results that Pum1 directly interacts with ATXN1 and influences its spontaneous aggregation process (Figure 5A). The expression of mCherry- and YFP-tagged fusion proteins in mammalian cells was also verified by SDS-PAGE and immunoblotting (Figure S12D and Figure S13).

### The protein MED15ΔCC does not promote YFP-ATXN1Q82 aggregation and toxicity in cell-based assays

**Figure 7:**
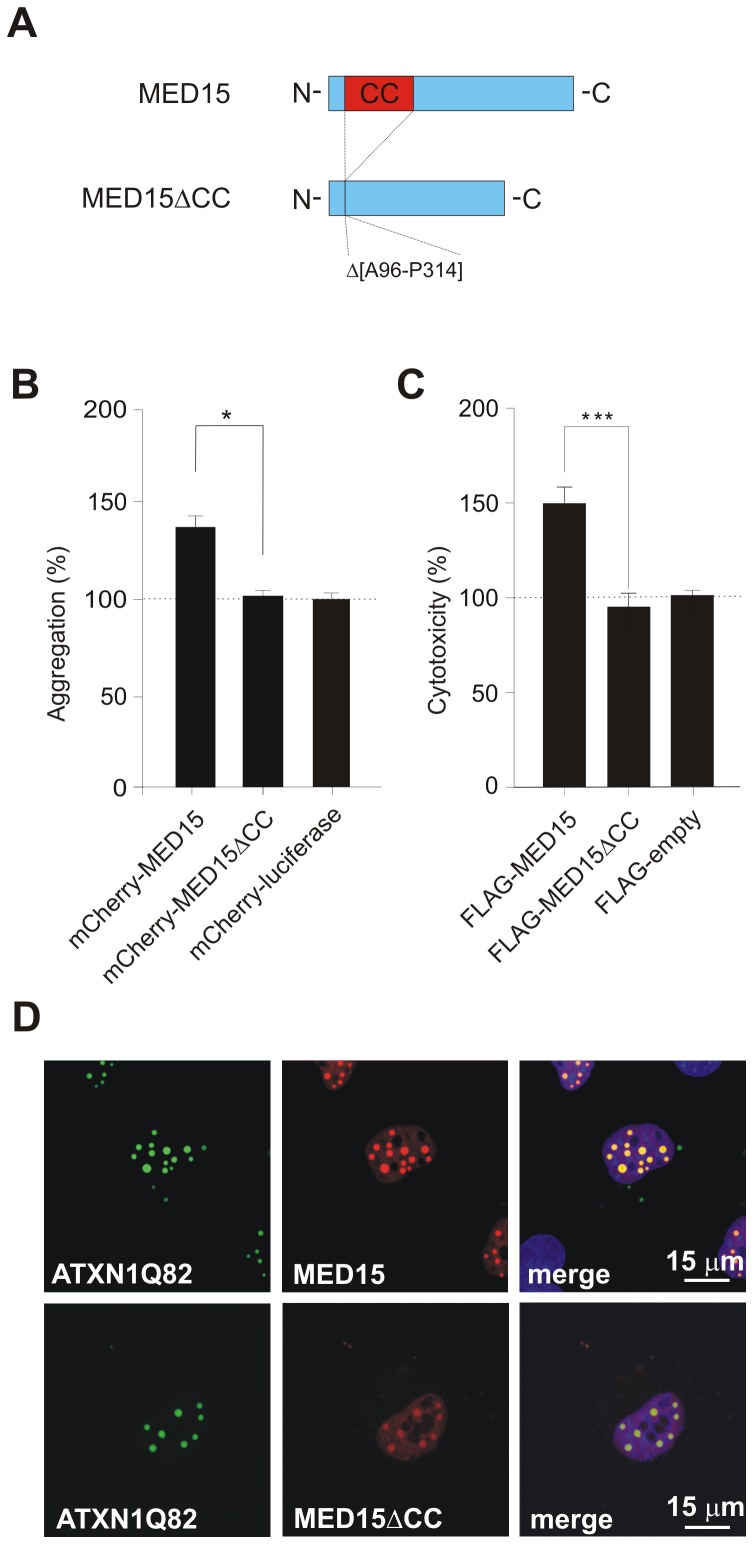
Effects of MED15ΔCC on mutant ATXN1 aggregation and cytotoxicity in cell-based assays. (A) Schematic representation of the MED15ΔCC fusion protein. (B) Effects of mCherry-tagged proteins MED15, MED15ΔCC and luciferase on spontaneous YFP-ATXN1Q82 aggregation in neuroblastoma SH-EP cells. The formation of YFP-ATXN1Q82 aggregates was quantified by fluorescence imaging after 48 h. Data were normalized to the total mCherry fluorescence intensities. Data is shown as mean ± SD from three independent experiments. Experiments were performed in triplicates. Student's t-test was used for statistical comparisons, p<0.05. (C) Effects of FLAG-tagged fusion proteins MED15 and MED15ΔCC on YFP-ATXN1Q82-induced cellular toxicity in COS-1 cells. YFP-ATXN1Q82 cytotoxicity was increased in the presence of full-length MED15 but not in the presence of the truncated fragment MED15ΔCC. Cells transfected with the control plasmid pFLAG were used as a control (100%). (D) Confocal microscopy images of COS-1 cells co-transfected with pairs of plasmids encoding YFP-ATXN1Q82 and mCherry-tagged MED15 or MED15ΔCC. Nuclei were stained with Hoechst 33342 (blue).

We also examined whether a truncated MED15 fragment lacking the N-terminal CC domain (MED15ΔCC) influences spontaneous ATXN1 aggregation (Figure 7A and 7B). The mCherry-tagged proteins MED15 and MED15ΔCC were co-produced with YFP-ATXN1Q82 in neuroblastoma cells and the formation of polyQ-containing protein aggregates was quantified after 48 h by high content fluorescence imaging [53]. We found that co-production of full-length MED15 increased the deposition of insoluble YFP-ATXN1Q82 aggregates in mammalian cells (Figure 7B), confirming the results in cell-free aggregation assays (Figure 5A). In contrast, YFP-ATXN1Q82 aggregation was not increased in cells co-producing the truncated protein MED15ΔCC, indicating that the N-terminal CC domain in MED15 indeed is critical for the stimulation of ATXN-1 aggregation.

Next, we used the established COS-1 cell model to investigate the effects of MED15 and MED15ΔCC on YFP-ATXN1Q82 toxicity. FLAG-tagged MED15 and MED15ΔCC fusion proteins were co-produced with YFP-ATXN1Q82 in COS-1 cells and after 48 h the caspase-3/7 activity was quantified in cell extracts. We found that YFP-ATXN1Q82 toxicity was significantly increased in the presence of full-length MED15 protein but not in the presence of the truncated fragment MED15ΔCC (Figure 7C), indicating that the CC domain in MED15 influences both aggregation and toxicity of mutant ATXN-1 in mammalian cells.

Finally, we examined whether the N-terminal CC domain in MED15 influences the association of the protein with YFP-ATXN1Q82 in mammalian cells using confocal microscopy (Figure 7D). We observed that full-length mCherry-tagged MED15 fusion protein readily co-localized with YFP-ATXN1Q82 aggregates in intranuclear inclusion bodies. However, co-localization was diminished with the mCherry-tagged protein MED15ΔCC, indicating that the N-terminal CC domain in MED15 is critical for the binding of the fusion protein to YFP-ATXN1Q82 (Figure 7D). The expression of mCherry- and FLAG-tagged fusion proteins in mammalian cells was confirmed by SDS-PAGE and immunoblotting (Figure S13).

## Discussion

### Cell-based assays detect ATXN1 toxicity and aggregation modifiers

In previous studies a large number of modifier proteins have been identified that influence misfolding or toxicity of polyQ disease proteins such as huntingtin, ataxin-1 or ataxin-3 [17], [19], [26]. These modifiers give important hints as to which cellular pathways are altered in polyQ diseases; however, they do not immediately allow a comprehensive mechanistic understanding of disease processes in neurons or patient brains. It often remains unclear whether modifiers identified in model organisms have a direct or indirect effect on misfolding and aggregation of polyQ disease proteins and whether human homologues of such proteins are also effective.

In this study, ∼200 human modifier proteins were predicted using available information from genetic screens in lower model organisms [55]. These proteins were systematically tested for their effects on the toxicity and aggregation of a pathogenic ATXN1 fragment (YFP-ATXN1Q82^NT^) in cell-based assays. Our investigations revealed 21 modulators that reproducibly enhanced or reduced the toxicity of YFP-ATXN1Q82^NT^ (Figure 1E). Most of these proteins (71.4%) also showed a strong effect on polyQ-mediated ATXN1 protein aggregation in filter retardation assays (Figure 1F), supporting previous findings that protein misfolding is associated with dysfunction and toxicity in mammalian cells [17], [24]. Experiments involving ATXN3, an unrelated polyQ disease protein [56], revealed that most toxicity modifiers were specific for YFP-ATXN1Q82^NT^, indicating that polyQ tracts, which are present in both ATXN1 and ATXN3, did not dominate modifier selection. Overall, our studies demonstrate that cell-based cDNA over-expression assays allow the identification of disease protein-specific toxicity and aggregation modifiers.

### Modulators of ATXN1 toxicity and aggregation play important functional roles in a variety of cellular processes

Using Gene Ontology information [34], we analyzed the annotated cellular functions of YFP-ATXN1Q82^NT^ toxicity modifiers. We found that proteins involved in protein translation, protein folding, RNA metabolism, membrane fusion and protein biosynthesis were overrepresented among modifiers. This is in agreement with previous observations from genetic screens in model organisms [55]. Our observations are also in line with the view that an interplay between different cellular pathways controls polyQ-mediated protein aggregation and proteotoxicity [57], [58], which balance protein biosynthesis, folding, translocation and clearance.

#### Protein folding modulators

Previous studies in disease model systems have demonstrated that overproduction of molecular chaperones such as Hsp70 or Hsp40 efficiently reduce the toxicity of polyQ disease proteins [22], [59]. This effect can be observed with chaperones in various species from bacteria to human [60], indicating that protein folding pathways are highly conserved and are a first line of cellular defense against the accumulation of toxic misfolded proteins in all organisms. Our studies support the thesis that molecular chaperones are key players in protein homeostasis networks. We found that overproduction of DNAJB4, a member of the Hsp40 chaperone family [61], efficiently reduced the toxicity of pathogenic YFP-ATXN1Q82^NT^ in cell-based assays. Interestingly, this effect was accompanied by an increase of SDS-insoluble ATXN1 aggregates, indicating that high levels of DNAJB4 reduce cellular toxicity but promote the assembly of polyQ-containing protein aggregates. A similar result was obtained with the mitochondrial chaperone AFG3L2 (Figure 1E and 1F). This protein belongs to the family of ATP dependent AAA proteases [62], which regulate the degradation of misfolded polypeptides in mitochondria. The importance of AFG3L2 in maintaining cellular protein function is underscored by the recent observation that mutations in this protein cause the neurodegenerative disease SCA28 [63], [64]. Thus, our data establish a potential link between the pathogenesis in SCA1 and SCA28 that needs to be further investigated genetically and mechanistically.

#### Modulators of RNA synthesis and processing

Our studies also suggest that some ATXN1 toxicity modifiers are RNA-binding proteins, which are functionally involved in the regulation of transcription and translation. For example, we identified the RNA-binding protein Pum1 as an ATXN1 toxicity modulator in cell-based assays. It functions as a repressor of translation and most probably influences dendrite morphogenesis and synaptic function in neurons [65]. Pumilio, the *Drosophila* homologue of human Pum1, was previously identified as a potent enhancer of ATXN1 toxicity in SCA1 transgenic flies [19]. In our cell-based assays, however, human Pum1 functioned as a suppressor of YFP-ATXN1Q82^NT^ toxicity (Figure 1E). We suggest that the CC domain, which can be computationally predicted in the fly but not in the human protein (Figure S7B), might be responsible for these opposing effects. CC domains are well known mediators of protein-protein interactions [66], [67], suggesting that the CC in Pumilio might function as a template that promotes the intermolecular association of aggregation-prone ATXN1 molecules. However, more detailed comparative studies with the fly and the human proteins are necessary to substantiate this hypothesis.

#### Modulators of protein translation

Proteins involved in translation were also overrepresented among ATXN1 toxicity modifiers in this study (adjusted p-value<0.05; Figure 3A). This includes ribosomal proteins such as P0 or L10 as well as regulators of protein synthesis such as EIF2G [68]. The identification of proteins that influence translation is not unexpected, as it is well known that protein levels are critical for aggregation and toxicity of polyQ disease proteins in cells [69]. Interestingly, the eukaryotic translation initiation factor subunit B (EIF2G) was identified as a potent suppressor of YFP-ATXN1Q82^NT^ toxicity. This protein is a component of the eukaryotic initiation factor 2 (eIF2), which mediates tRNA^met^ binding to ribosomes and controls global protein synthesis [70]. Previous studies have demonstrated that stress kinases such as PKR, which are activated in brains of patients with neurodegenerative diseases [71], can inactivate eIF2 function through phosphorylation. This leads to a reduction in protein synthesis and the activation of cell death pathways [72]. Our results suggest that the toxicity suppressing effect of EIF2G in cells with YFP-ATXN1Q82^NT^ might be due to a re-activation of eIF2 function, leading to improved protein translation and reduced apoptosis.

#### Modulators of protein and vesicle trafficking

Our cell-based toxicity assays also identified several modifiers with important functions in protein and vesicle transport processes (Table S4). This was not expected from previous modifier studies, which showed that mainly molecular chaperones, RNA binding proteins and transcription regulators influence the toxicity of pathogenic ATXN1 or ATXN3 in lower model organisms [55]. We found, e.g., that the vacuolar sorting associated protein Vps4B is a potent modulator of polyQ toxicity in cell-based assays. Vps4B is an AAA ATPase mediating the transport of proteins from endosomes to lysosomes [73]. Its function is tightly linked to the endosomal sorting complex required for transport machinery (ESCRT), a large membrane-associated protein complex, which is also critical for efficient autophagy-mediated degradation of misfolded proteins [74], [75]. Recent studies indicate that mutations in ESCRT proteins such as CHMP2B can cause neurodegeneration and the accumulation of misfolded proteins in neuronal cells [75], [76], supporting our observations that proteins with key functions in vesicle transport processes influence aggregation and toxicity of mutant ATXN1.

### Structural characteristics of ATXN1 toxicity modifier proteins

Recent studies indicate that alpha-helical coiled-coil (CC) domains are critical for the spontaneous aggregation of Q/N-rich yeast prions and polyQ disease proteins [39], [40]. This suggests that such domains, known to promote protein-protein interactions [67], might also be present in modifier proteins and contribute to their effects on ATXN1 aggregation and toxicity in cell-based assays. We computationally predicted that 6 of 21 YFP-ATXN1Q82^NT^ toxicity modifiers contain CC domains (Table S5). In addition, we found that CC domains are exclusively present in ATXN1 toxicity and aggregation enhancers (Figure 3B), suggesting that they are critical for this effect in mammalian cells. We hypothesized that proteins with CC domains may enhance protein aggregation and toxicity because they directly promote the intermolecular association of ATXN1 molecules. To test this hypothesis, we purified the ATXN1 interacting proteins MED15 and Pum1 and investigated whether they influence polyQ-mediated ATXN1 aggregation in cell-free assays. We predicted that MED15 contains a large N-terminal CC domain (Figure S7A), while such a domain was not detectable in the Pum1 protein (Figure S7B). However, both proteins were found to interact with ATXN1 in cell-based co-immunoprecipitation assays (Figure 4B). *In vitro* aggregation studies with recombinant, purified proteins revealed that the CC domain-containing protein MED15 stimulates spontaneous ATXN1Q82 aggregation, while such an effect was not observed with the protein Pum1 lacking a CC domain (Figure 5A and 5B).

Using CD spectroscopy, we confirmed that the computationally predicted N-terminal CC domain in MED15 (MED15CC, aa 141–316) adopts a typical α-helical CC structure (Figure 5D). Moreover, we examined whether this domain alone is sufficient to promote ATXN1Q82 misfolding in cell-free aggregation assays. We found that MED15CC similar to full-length MED15 is a potent enhancer of polyQ-mediated protein aggregation *in vitro*, supporting the hypothesis that this N-terminal domain is critical for MED15-mediated stimulation of ATXN1 aggregation (Figure 5E and Figure 5F). We also examined whether a hybrid Pum1 protein with an N-terminal MED15CC fragment can promote ATXN1 aggregation in cell-based assays. We observed that in strong contrast to the wild-type Pum1 protein the hybrid Pum1-MED15CC fusion is a potent ATXN1 aggregation enhancer (Figure 6B and Figure S12A). Finally, we investigated whether a truncated MED15 fragment lacking the N-terminal CC domain (MED15ΔCC) influences mutant ATXN1 aggregation and toxicity in cell-based assays. We observed that MED15ΔCC in contrast to the wild-type protein does not promote ATXN1 aggregation and toxicity (Figure 7B and 7C). Thus, our cell-based studies support the results obtained in cell-free assays, indicating that the N-terminal α-helical CC region in MED15 is critical for efficient stimulation of spontaneous protein aggregation [39].

Several lines of experimental evidence indicate that long polyQ tracts in neurodegenerative disease proteins such as ATXN1 have to undergo a conformational change before they can assemble into insoluble protein aggregates [77], [78]. While polyQ tracts in soluble proteins often have a random coil or an α-helical conformation, they adopt a characteristic β-sheet structure in insoluble protein aggregates [79]. Thus, it is currently thought that a conformational switch from an α-helical to a β-sheet conformation controls spontaneous polyQ-mediated protein aggregation [80]. Our experiments indicate that interacting proteins with CC domains such as MED15 can promote the spontaneous aggregation of polyQ disease proteins, because they function as a template for the efficient transition of helical domains to β-sheets. This view is also supported by previous investigations, indicating that α-helical structures are critical intermediates in the aggregation pathway of β-sheet-rich amyloid structures [81]. We suggest that this mechanism may have important implications for the development of therapeutic strategies against polyQ diseases, because interaction partners with CC domains are potent enhancers of abnormal protein aggregation. Thus, higher concentrations of proteins with CC domains in neurons might initiate the disease process, causing progressive dysfunction of the cellular proteostasis network [82]. In the long run, this might lead to cellular degeneration and the activation of cell death pathways [57], [82]. However, more detailed studies investigating the potential dysregulation of CC domain-containing proteins in neurons are necessary to fully understand the impact of CC proteins on polyQ aggregation and toxicity in cells.

### Conclusions

Taken as a whole, our study shows that a strategy combining bioinformatic and experimental approaches is useful for the identification of human modifier proteins that influence the toxicity and aggregation of pathogenic ATXN1. These modifiers play a functional role in a variety of different cellular mechanisms that are currently not very well understood. Further investigations with human proteins and their homologues from lower model organisms such as *D. melanogaster* or *C. elegans* are necessary to elucidate the cellular processes that increase or reduce the toxicity of pathogenic polyQ proteins. We suggest that a step-by-step process of data integration and refinement as well as dissection of molecular details in signaling cascades and cellular protein quality control pathways will lead to the discovery of new drug targets and therapeutic strategies, which can be tested in appropriate disease models and patients with polyQ diseases.

## Materials and Methods

### Generation of Gateway entry plasmids

cDNA fragments encoding human proteins MED15CC (M141-S316, 175 aa), ATXN1Q30 (818 aa), ATXN1Q82 (868 aa), ATXN1Q30^NT^ (458 aa), ATXN1Q82^NT^ (508 aa), ATXN3Q25 (360 aa) and ATXN3Q73 (408 aa) were PCR amplified with Pwo SuperYield DNA polymerase (Roche) from the plasmids pOTB7-MED15 (cloneID:CCSB7005), pAS2-1-ATAXIN1Q30, pAS2-1-ATAXIN1Q82, pBTM116-ATXN3Q25 and pBTM116-ATXN3Q73 and cloned into the Gateway plasmid pDONR221 (Invitrogen). Oligonucleotides (MED15CC-forward, MED15CC-reverse, ATXN1-forward, ATXN1-reverse-FR, ATXN1-reverse-FL, ATXN3-forward, and ATXN3-reverse, Table S6) were purchased from BioTez Berlin-Buch GmbH, Germany. PCR reactions (50 µl volume) contained 50 ng plasmid DNA, 100 pmol primer oligonucleotides, 2 mM MgCl_2_, 50 mM KCl, 10 mM Tris–HCl pH 8.0, 0.1% Triton-X100 and 2.5 U Pwo DNA polymerase. Fragments were amplified in 30 cycles with the following profile: 45 s denaturation at 93°C, 45 s annealing at 52°C and 3 min extension at 72°C. Amplified 589 bp DNA fragments were isolated from agarose gels and combined with the pDONR221 plasmid (Invitrogen) at a 1∶2 ratio in the presence of BP clonase (Invitrogen). After incubation for 16 h at 25°C the resulting entry DNA plasmids were transformed into competent Mach1 *E. coli* cells (Invitrogen). *E. coli* clones were selected on LB agar plates supplemented with Kanamycin and analyzed by BsrGI restriction digestion (NEB). Finally, the identity of DNA fragments was verified by DNA sequencing. The pJET-MED15CC plasmid was generated by isolating the 554 bp PCR product from an agarose gel and blunt end cloning into the pJET1.2 vector (Fermentas) according to the manufacturer's recommendations. Identity of the DNA fragment was verified by DNA sequencing. To create the Gateway compatible entry plasmid encoding Pum1-MED15CC (pePUM1-MED15CC), the 537 bp cDNA fragment MED15CC was subcloned into the unique AatII site in the Gateway compatible plasmid pOBT7-Pum1 (cloneID:CCSB6127) encoding the Pum1 protein (Figure 6A). The correct insertion of the cDNA fragment was confirmed by restriction digestion. To create the Gateway compatible entry plasmid (pe-MED15ΔCC) encoding MED15ΔCC, the plasmid sequence of pOTB7-MED15 (cloneID: CCSB7005) was amplified by PCR without the insert encoding the N-terminal MED15CC domain (A96-P314) using MED15ΔCC_Fwd and MED15ΔCC_Rev primers (Table S6). For amplification of the linear plasmid DNA we used the Phusion Site-Directed Mutagenesis Kit (Finnzymes) following the recommended protocol. The resulting MED15ΔCC cDNA was verified by DNA sequencing.

### Generation of expression plasmids

For cell-based modifier screens, we cloned 202 computationally selected genes (Table S7) into the plasmids pFLAG-GW and pACT4-DM (Invitrogen), respectively. The cDNA fragment encoding ATXN1Q82^NT^ was cloned into the pdEYFP-C1Amp vector (Imagenes). cDNA fragments encoding ATXN1Q30 and ATXN1Q82 were cloned into the vector pPA-Reni-DM [45] for LUMIER interaction assays. cDNAs encoding Pum1, MED15 and U2AF2 were also subcloned into the plasmid pFire-V5-DM [45]. Mammalian expression plasmids were generated by subcloning of cDNAs encoding the proteins MED15, MED15ΔCC, Pum1, Pum1-MED15CC, MED15CC, STUB1 and S100B into the plasmids pmCherryGW (Invitrogen) and pFLAG-GW (Invitrogen). To produce proteins in *E. coli*, the cDNA fragments encoding ATXN1Q82 and MED15CC were cloned into the expression vector pGEX-6P-1 (Amersham Biosciences); cDNA fragments encoding the proteins MED15 and Pum1 were subcloned into the plasmid pDESTCo (Invitrogen). For generation of destination plasmids the Gateway recombination cloning technology was applied as recommended by the manufacturer (Invitrogen). Entry plasmids and empty destination vectors (Table S8) were combined in a 1∶3 ratio in the presence of LR clonase (Invitrogen) and subsequently incubated for 1 h at 25°C. Then, plasmids were transformed into competent Mach1 *E. coli* cells (Invitrogen) as recommended by the manufacturer. Finally, plasmid DNAs were purified from *E. coli* clones using the QIAprep 96 Turbo Miniprep Kit (Qiagen) and analyzed by BsrGI (NEB) restriction digestion.

### Overproduction of recombinant proteins in COS-1 cells

COS-1 cells were grown to 70–80% confluency in Dulbecco's modified Eagle medium (DMEM) supplemented with 5% fetal calf serum, penicillin (100 µg/ml) and streptomycin (100 µg/ml). For cDNA overexpression experiments, cells at passage 5–6 were seeded into 96-well cell culture plates (12,000 cells/well). Then, they were co-transfected with pdEYFP-C1Amp (0.4 µg) and a pFLAG-GW-based plasmid (0.4 µg) encoding e.g., the proteins YFP-ATXN1Q82^NT^ and the FLAG-tagged modifier protein MED15. Transfections were performed using Lipofectamine 2,000 in Opti-MEM (Invitrogen). In each co-transfection experiment ∼200 putative modifiers were systematically tested. All co-transfection experiments were performed in triplicates. Transfected COS-1 cells were harvested after 48 h and lysed in 1% NP-40 PBS, supplemented with a complete protease inhibitor cocktail mix (Sigma). To separate the supernatant from the insoluble pellet, crude cell extracts were centrifuged at 30,000 rpm for 30 min. The pellet was dissolved in 8 M urea.

### Antibodies

To generate a polyclonal antibody against ATXN1, two peptides (aa11–26, CLPPKKREIPATSRSS and aa326–338, GEMEKSRRYGAPS) were independently fused to the carrier Keyhole Limpet Hemocyanin (KLH) protein and injected into rabbits using standard immunization procedures (Eurogentec, Belgium). The resulting immune sera were affinity purified against His-tagged ATXN1Q30 immobilized on a Ni-NTA column (Qiagen), according to manufacturer's instructions. The specificity of antibodies was confirmed by SDS-PAGE and immunoblotting using protein extracts from transiently transfected COS-1 cells (Figure S14). The anti-ATXN1 antibody SA4645 was used for the detection of full-length ATXN1 and ATXN1Q82^NT^. The mouse monoclonal anti-FLAG antibody M2 (Sigma-Aldrich) was used (1∶1,000) for the detection of FLAG-tagged modifier proteins produced in COS-1 cells. Recombinant ATXN3Q25 and ATXN3Q73 proteins were detected with the anti-MJD CT antibody (1∶1,000) [83]. To monitor mCherry-tagged recombinant fusion proteins, Living Colors mCherry monoclonal antibody (1∶1,000, Clontech) was used.

### siRNA knock down of target genes in COS-1 cells

For siRNA assays, COS-1 cells were transfected with 0.2 µg of a pdEYFP-C1Amp vector encoding YFP-ATXN1Q82^NT^ and 2 pmol target gene siRNA in 24 well plates (50,000 cells per well). siRNAs were obtained from the HP GenomeWide siRNA library (Qiagen). The cell culture conditions and transfection reagents were as described in protein overproduction experiments. To confirm the knock-down of target genes qRT-PCR experiments were performed. RNA was purified from cells using the RNeasy Mini Kit (Qiagen) as recommended by the manufacturer. cDNA generation and qRT-PCR reactions were performed using the Dynamo FLASH SYBR Green qPCR kit (Finnzymes) in an Applied Biosystems 7,500 operating system. Each experiment was performed in triplicate. The sequences of oligonucleotides used in qPCR experiments are displayed in Table S9.

### Caspase 3/7 toxicity assays

An equal number of COS-1 cells (12,000 cells per well) were co-transfected with plasmids encoding YFP-ATXN1Q82^NT^ and a potential FLAG-tagged modifier protein. Caspase 3/7 activity in transfected COS-1 cells was measured using the Apo-ONE Homogeneous Caspase-3/7 Assay kit (Promega). Briefly, cell lysis buffer was mixed with a pre-fluorescent caspase 3/7 substrate and added to COS-1 cells 48 h after transfection. The pre-fluorescent caspase 3/7 substrate was cleaved by activated caspase 3/7 and resulted in the formation of a fluorescent product. Fluorescence was measured in a plate reader (TECAN Infinite M200) and caspase 3/7 activity was normalized to the cell numbers quantified via high content imaging microscopy (Thermo Scientific, Arrayscan VTI). Each experiment was performed in triplicates.

### Filter retardation assays

For quantification of insoluble polyQ-containing protein aggregates a filter retardation assay was applied [32]. COS-1 cells were transfected as described in the previous paragraph. Equal amounts of crude cell extracts (120 µg total protein) were mixed with an equal volume of 4% SDS and 100 mM DTT and heated at 95°C for 5 min. Samples were diluted in 100 µl 0.2% SDS and filtered through a 0.2 µm cellulose acetate membrane. SDS-resistant aggregates retained on the membrane were detected using the polyclonal anti-ATXN1 antibody SA4645 (1∶1,000).

### Quantification of insoluble polyQ-containing protein aggregates by high-content fluorescence imaging

SH-EP cells were grown at 37°C and 5% CO_2_ in Dulbecco's modified Eagle medium (DMEM) supplemented with 4.5 g/l D-glucose, 10% fetal calf serum, penicillin (100 µg/ml) and streptomycin (100 µg/ml). Cells were seeded in 96-well cell culture plates (10,000 cells/well) and co-transfected with a 1∶1 mix of cDNAs encoding YFP-ATXN1Q82 or YFP-ATXN1Q82^NT^ and mCherry-tagged modifiers (200 ng each). 48 h after transfection cells were fixed with 4% paraformaldehyde (Sigma) and DNA was stained with Hoechst 33342 (1∶2,500) (Invitrogen). The cell culture plates were then placed into a high-content screening (HCS) cell analysis system (Arrayscan VTI, Thermo Scientific). The principle of HCS analysis is described elsewhere [84]. Nuclei were identified by Hoechst fluorescence; cell dimensions were fitted using the ArrayScan VTI software (Thermo Scientific). ATXN1Q82, ATXN1Q82^NT^ and ATXN3 overproduction was quantified by YFP fluorescence and modifiers were monitored by mCherry fluorescence. The fluorescence signals were normalized to cell numbers. Experiments were performed in triplicate.

### Confocal microscopy of YFP-ATXN1Q82 and -ATXN1Q82^NT^ aggregates and modifier proteins in COS-1 cells

COS-1 cells were grown at 37°C and 5% CO_2_ in Dulbecco's modified Eagle medium (DMEM) supplemented with 4.5 g/l D-glucose, 10% fetal calf serum, penicillin (100 µg/ml) and streptomycin (100 µg/ml). Cells were seeded on coverslips in 24-well cell culture plates (25,000 cells/well). Co-transfections were performed using cDNAs encoding YFP-ATXN1Q82, YFP-ATXN1Q82^NT^ and mCherry-tagged modifiers (500 ng each). For single transfections cells were transfected with cDNAs encoding YFP-ATXN1Q30, YFP-ATXN1Q82, YFP-ATXN1Q30^NT^ or YFP-ATXN1Q82^NT^ (500 ng each). 48 h after transfection cells were fixed with 4% paraformaldehyde (Sigma), stained with Hoechst 33342 (1∶2,500 in PBS, Invitrogen) and mounted using ProLong Gold antifade reagent (Invitrogen). ATXN1Q82 and ATXN1Q82^NT^ overproduction was monitored by YFP fluorescence and modifiers were detected by mCherry fluorescence. Images were recorded using a Leica SP2 confocal microscope.

### Knock-down of target genes in SCA1 transgenic flies

For knock-down of niki and MED15 gene expression the VDRC stocks 16,120 and 21,809 were used (http://stockcenter.vdrc.at/control/main). UAS-GFP and UAS-GFP-RNAi lines, provided by C. Antoniewski were used as controls. J. Botas and M. O. Fauvarque kindly provided the fly lines harbouring UAS-ATXN1Q82 and GMR-GAL4 genes. Initially, a stable UAS-ATXN1Q82;GMR-GAL4 line was generated. To analyze the effects of niki and MED15 target gene knock-downs on ATXN1Q82 induced eye degeneration UAS-ATXN1Q82;GMR-GAL4 females were crossed with males of niki and MED15 RNAi lines and with males of UAS-GFP and UAS-GFP-RNAi control lines, respectively. The eye structure of male progeny (n>20) was examined using Leica and Zeiss stereomicroscopes. For electron microscopy imaging, flies (n>5) were desiccated at 40°C overnight, dried by the critical point method with liquid N_2_, sputter-coated with gold and visualized with a scanning electron microscope (JEOL JSM 840-A).

### Detection of protein–protein interactions

For LUMIER assays protein A (PA)-Renilla luciferase (RL)-tagged ATXN1 fusion proteins (PA-RL-ATXN1Q30 or PA-RL-ATXN1Q82) were co-produced with firefly luciferase-tagged modulator proteins FL-Pum1, FL-MED15 or FL-U2AF2 in HEK293 cells. After 48 h protein complexes were co-immunoprecipitated from cell extracts with IgG coated magnetic beads (Dynabeads; Invitrogen); interactions between bait (PA-RL fusions) and prey proteins (FL fusions) were monitored by quantification of firefly luciferase activities [45]. Quantification of Renilla luciferase activity was used to confirm that PA-RL-tagged bait proteins are successfully immunoprecipitated from cell extracts. To detect Renilla and firefly luciferase based luminescence in samples the Dual-Glo Luciferase Kit (Promega) was used. Bioluminescence was quantified in a luminescence plate reader (TECAN Infinite M1000). A representative example of a typical LUMIER-based co-immunoprecipitation experiment with transiently transfected HEK293 cells is shown schematically in Figure 4A and Figure S8. It is important to note that for each protein pair that was investigated (interaction between selected bait and prey proteins) three co-immunoprecipitation experiments (Co-IPs A–C) were performed in parallel in HEK293 cells, in order to assess the specificity of an interaction. For example, to investigate the interaction between the proteins ATXN1Q30 and Pum1 the protein pairs (A) PA-RL-ATXN1Q30/FL-Pum1, (B) PA-RL/FL-Pum1 and (C) PA-RL-ATXN1Q30/FL are individually co-produced in HEK293 cells (Figure S8). The proteins PA-RL and FL in experiments B and C are used as controls to examine background protein binding. The resulting protein complexes in Co-IPs A–C are systematically analyzed by quantification of firefly luciferase activity. By dividing the firefly luminescence activity measured in sample A through activities found in samples B and C the R-op and R-ob binding ratios are obtained, which function as a measure for the protein interaction specificity. Low R-op values indicate unspecific prey protein interactions, while low R-ob values indicate unspecific bait protein interactions (Figure S8). Based on empirical studies with a set of well-characterized positive and negative interaction pairs (not shown), we defined that R-op and R-ob binding ratios of >1.5 are indicative of reliable protein-protein interactions.

### Protein purification and aggregation assays

For the production of GST- and His-tagged fusion proteins the genes encoding the proteins ATXN1Q82, MED15CC, MED15 and Pum1 were cloned into the vectors pGEX-6P-1 and pDEST-Co and the resulting plasmids pGEX-6P-1-ATXN1Q82, pGEX-6P-1-MED15CC, pDEST-Co-MED15 and pDEST-Co-Pum1 were transformed into *E. coli* BL21 cells. Recombinant proteins were purified using glutathione agarose (Sigma) or Ni-NTA agarose (Qiagen) beads according to the instructions of manufacturers. His-tagged Pum1 protein was purified under denaturing conditions using 6 M guanidium-HCl and refolded in buffer A (500 mM (NH_4_)_2_SO_4_, 50 mM MgCl_2_, 10 mM DTT, 30% glycerol) for 16 h on ice. The supernatant was dialyzed against buffer B (10 mM Tris-Cl pH 8, 100 mM NaCl, 1 mM EDTA, 10% glycerol) for 16 h at 4°C. His-Pum1 protein was then concentrated using mini spin-columns (Millipore).

For *in vitro* aggregation assays, GST-ATXN1Q82 (5–7 µM) was incubated with His-tagged MED15, His-tagged Pum1 or GST-tagged MED15CC in the presence of PreScission Protease (Amersham Pharmacia) at 30°C with constant shaking (300 rpm). Reactions were stopped by freezing of samples in liquid nitrogen. 25 µg of GST-ATXN1Q82 was filtered through a 0.2 µm cellulose acetate membrane and protein aggregates retained on filter membranes were detected using the polyclonal anti-ATXN1 antibody SA4645 (1∶1,000).

### Circular dichroism spectroscopy

GST and GST-MED15CC proteins were dialyzed in 50 mM Na_2_HPO_4_/NaH_2_PO_4_, 150 mM NaF pH 7.5 for 16 h at 4°C. Far-UV circular dichroism spectra of GST and GST-MED15CC (5 µM) were recorded at 20°C using a Chirascan CD spectrometer (Applied Biophysics) at a bandwidth of 1 nm. Spectra were collected at 1 nm intervals from 250 to 190 nm and at least 3 average values for each measurement were smoothed and normalized to mean residual weight ellipticity [θ] using the Pro-Data software (Applied Biophysics). MED15CC CD values were acquired by substraction of the GST CD spectrum from the GST-MED15CC CD spectrum. Prediction of secondary protein structure based on the CD data was performed using the K2D2 program [49].

### Bioinformatics analysis

The EASE program [34] was used to identify over-represented functional categories (Gene Ontology annotation and KEGG pathways) among the identified 21 YFP-ATXN1Q82^NT^ toxicity modifiers. The modifier genes were compared to the human genome; a Fisher's exact test was performed to identify over-represented functional categories. We only considered categories with adjusted p-values<0.05 (adjusted with the Bonferroni method) as statistically significant. For the prediction of coiled-coil domains (CC) in amino acid sequences of modifier proteins the COILS program was used [41]. Only high probability CC sequences (0.8–1) were considered for further investigations. We used Chi-square tests to compare the number of YFP-ATXN1Q82^NT^ toxicity modifiers with CC domains to the human proteome (73,427 protein sequences in SwissProt DB, 20.3%).

To identify glutamine-rich (Q-rich) or polyglutamine (polyQ) proteins, a window of 50 amino acids was passed over each protein sequence. For each window position the fraction of Qs amongst all residues or the number of consecutive Qs was counted. A protein was considered as Q-rich when it contains >50% Qs in a stretch of at least 10 amino acids; in comparison a protein was defined as a polyQ protein when it contains a stretch of >10 consecutive Qs.

Sequence homologues of human Pum1 and MED15 were selected using the NCBI BLAST sequence search program [28]. For multiple sequence alignments we used the EBI's MUSCLE web tool [85]. The local glutamine composition of aligned sequences was determined by shifting a window of 50 amino acids over all sequences and by counting the fraction of Qs amongst all residues for each window position (not considering gaps). Additionally, the fraction of Qs in an uninterrupted polyQ stretch (>5) was counted for each protein. This information was used to compare the Q and polyQ content of homologous proteins.

## Supporting Information

Figure S1 Western blot analysis of FLAG-tagged modulator proteins. Plasmids encoding FLAG-tagged modifier proteins were randomly selected and transfected into COS-1 cells. Protein extracts were prepared after 48 h and analyzed by SDS-PAGE and immunoblotting using the anti-FLAG antibody M2. The same amount of total protein was loaded in each lane.
(TIF)

Figure S2 Detection and quantification of YFP-ATXN1Q30^NT^ or YFP-ATXN1Q82^NT^ aggregates in COS-1 cells. (A) A schematic representation of full-length and truncated ATXN1 proteins with pathogenic and non-pathogenic polyQ tracts. (B) Confocal microscopy pictures of YFP-ATXN1Q82^NT^ and YFP-ATXN1Q30^NT^ protein aggregates detected in COS-1 cells. Cells were analyzed after 48 h. Nuclei are stained with Hoechst 33342 (blue) and YFP-ATXN1Q82^NT^ and YFP-ATXN1Q30^NT^ protein deposits are shown in green. (C) Quantification of YFP-ATXN1Q30^NT^ and YFP-ATXN1Q82^NT^ aggregates in COS-1 cells using a high-content screening cell analysis system (Arrayscan VTI, Thermo Scientific).
(TIF)

Figure S3 Schematic overview of modifier cytotoxicity screening strategy. COS-1 cells were co-transfected with pairs of plasmids encoding YFP-ATXN1Q82^NT^ and a potential toxicity modifier. Human proteins that either enhance (green) or suppress (red) the toxicity of pathogenic YFP-ATXN1Q82^NT^ are identified in a cell-based screen and subsequently retested in cells in the absence YFP-ATXN1Q82^NT^ fusion protein. Modulators were only considered for further investigations when they specifically influence YFP-ATXN1Q82^NT^ cytotoxicity in cell-based assays.
(TIF)

Figure S4 Western blot analysis confirms the production of YFP-ATXNQ82^NT^ toxicity modifiers in COS-1 cells. Overproduction of ATXN1Q82^NT^ cytotoxicity modifiers in transiently transfected COS-1 cells was analyzed after 48 h by SDS-PAGE and immunoblotting using the anti-FLAG antibody M2. Asterisks indicate modifier proteins.
(TIF)

Figure S5 Effects of human proteins on pathogenic ATXN3 cytotoxicity and aggregation. (A) Relative caspase 3/7 activity change induced by overproduction of proteins YFP-ATXN3Q25 or YFP-ATXN3Q73 in COS-1 cells compared to YFP overproducing cells. Caspase 3/7 activity was significantly increased in cells producing the ATXN3 fusion protein with a pathogenic polyQ tract compared to the wild-type protein (Student's t-test, ** p<0.01, n = 3). Error bars indicate SD. (B) Detection of SDS insoluble protein aggregates by filter retardation assay. The YFP-ATXN3Q73 fusion protein forms SDS-insoluble protein aggregates. Equal amounts of total protein were loaded. (C) Effects of modifier proteins on YFP-ATXN3Q73 cytotoxicity; 21 previously identified YFP-ATXN1Q82^NT^ toxicity modifiers (Figure 1E) were systematically tested in cell-based assays. We found that only the proteins P0 (RPLP0), Vps4B (VPS4B), Ran (RAN) and Nek8 (NEK8) influence YFP-ATXN3Q73 toxicity. (D) Effects of modifier proteins on YFP-ATXN3Q73 aggregation; overproduction of Ran and Vps4B enhanced YFP-ATXN3Q73 aggregation.
(TIF)

Figure S6 siRNA treatment reduces the expression of modifier genes in mammalian cells. siRNA treated COS-1 cells were analyzed by qRT-PCR. siRNA knock-down of six randomly selected target genes caused a reduction of mRNA levels by 50–90% in comparison to the non-targeting control. mRNA levels were normalized to the mRNA levels of β-actin. Data is shown as mean ± SD for three independent experiments performed in triplicates.
(TIF)

Figure S7 Prediction of coiled-coil domains in related MED15 and Pum1 proteins. Coiled-coil domains in proteins were predicted using the COILS program with a window size of 28 amino acids. (A) Predicted CC domain of human MED15 and its orthologues. (B) Predicted CC domain of human Pum1 and its orthologues. Abbreviations: hs, *Homo sapiens*; mm, *Mus musculus*; dm, *Drosophila melanogaster*; ce, *Caenorhabditis elegans*.
(TIF)

Figure S8 Schematic representation of a cell-based LUMIER co-immunoprecipitation assay. To investigate the interaction between the proteins ATXN1Q30 (bait) and Pum1 (prey) the fusion protein pairs (A) PA-RL-ATXN1Q30/FL-Pum1, (B) PA-RL/FL-Pum1 and (C) PA-RL-ATXN1Q30/FL were co-produced in HEK293 cells. After 48 h the cells were lysed and protein extracts were applied to IgG coated magnetic beads (Dynabeads; Invitrogen) for co-immunoprecipitation (co-IP) of interacting proteins. Following the co-IP the firefly luminescence signal was determined for all three samples (A–C) using a luminescence plate reader. In sample A, as a result of the interaction between PA-RL-ATXN1Q30 and FL-Pum1 a high luminescence signal was detected. In comparison, relatively low signals were obtained in control samples B and C. Using the measured values we calculated the binding ratios R-op and R-ob, which are measure for the specificity of the ATXN1Q30 and Pum1 interaction. Based on previous empirical studies with well-characterized interaction test pairs, we define an interaction as positive when the calculated R-op and R-ob ratios are >1.5.
(TIF)

Figure S9 Detection of firefly luciferase and Renilla luciferase tagged fusion proteins in HEK293 cell extracts. Production of Renilla and firefly luciferase-tagged fusion proteins in HEK293 cells was monitored by quantification of luminescence activity of protein extracts. Data are shown as mean ± SD for three separate experiments performed in triplicates. Abbreviations: FL - firefly luciferase; RL - Renilla luciferase; PA - protein A; RLU - relative light units.
(TIF)

Figure S10 Purification of recombinant GST- and His-tagged fusion proteins for cell-free aggregation assays. (A) Schematic representation of a spontaneous ATXN1Q82 aggregation reaction in the presence of a modifier protein. Aggregation of ATXN1Q82 is initiated by proteolytic cleavage of the fusion protein GST-ATXN1Q82 with PreScission protease. Insoluble ATXN1Q82 aggregates form spontaneously over time and are quantified by filter retardation assay. The effects of modifiers can be assessed in a time and concentration dependent manner. (B) GST- and His-tagged fusion proteins were produced in *E. coli* and purified by affinity chromatography. Analysis by SDS-PAGE and Coomassie staining confirmed that recombinant fusion proteins with expected sizes are produced in *E. coli*.
(TIF)

Figure S11 The recombinant proteins His-MED15 and His-Pum1 influence spontaneous ATXN1Q82 aggregation in a concentration-dependent manner. (A) Detection of SDS-insoluble ATXN1Q82 aggregates by filter retardation assay. GST-ATXN1Q82 (5 µM) was incubated with PreScission protease and modulator proteins (2.5, 5 or 10 µM) and formation of insoluble ATXN1Q82 protein aggregates was quantified after 48 h by filter assay. His-MED15 in a concentration dependent manner increased spontaneous ATXN1Q82 aggregation, while His-Pum1 has the opposite effect. SDS-insoluble ATXN1Q82 protein aggregates retained on filter membranes were detected using the anti-ATXN1 antibody SA4645. (B) Quantification of filter retardation assay data with the AIDA densitometry software. Error bars represent SD of three independent experiments.
(TIF)

Figure S12 Effects of modulator proteins on aggregation of full-length YFP- ATXN1Q82. (A) Effects of mCherry-tagged proteins Pum1-MED15CC, Pum1, MED15, S100B, STUB1 and luciferase on spontaneous YFP-ATXN1Q82 aggregation in human neuroblastoma SH-EP cells. The formation of polyQ-containing ATXN1 aggregates was quantified by fluorescence imaging after 48 h. We found that the hybrid protein Pum1-MED15CC in comparison to the wild-type Pum1 and the luciferase control protein (100%) readily promotes YFP-ATXN1Q82 aggregation, confirming the results obtained with the YFP-ATXN1Q82^NT^ protein (Figure 6B). An increase of YFP-ATXN1Q82 aggregates was also observed in MED15 and STUB1 overproducing SH-EP cells. In strong contrast, a suppression of spontaneous YFP-ATXN1Q82 aggregation was detected with the control protein S100B. Data are shown as mean ± SD from three independent experiments. Experiments were performed in triplicates. Student's t-test was used for statistical comparisons, p<0.05. (B) Analysis of spontaneous YFP-ATXN1Q30 and YFP-ATXN1Q82 aggregation in COS-1 cells by confocal microscopy. COS-1 cells were transfected with plasmids encoding the full-length ATXN1 proteins YFP-ATXN1Q30 and YFP-ATXN1Q82 and after 48 h cells were investigated by confocal microscopy. Nuclei were stained with Hoechst (blue). We found that both wild-type and mutant ATXN1 fusion proteins form nuclear inclusions in COS-1 cells (green). (C) Quantification of aggregate number and size in YFP-ATXN1Q30 and YFP-ATXN1Q82 overproducing cells a high-content screening cell analysis system (Arrayscan VTI, Thermo Scientific). (D) Detection of YFP-ATXN1Q30 and YFP-ATXN1Q82 fusion proteins in COS-1 cell extracts by SDS-PAGE and immunoblotting. Proteins were detected using anti-GFP and anti-actin antibodies. Abbreviations: TL - total lysate; P – pellet; Sup – supernatant.
(TIF)

Figure S13 Production of mCherry- and FLAG-tagged modifier proteins in mammalian cells. SH-EP neuroblastoma cells were transfected with plasmids encoding (A) mCherry- or (B) FLAG-tagged modifier proteins; protein extracts were analyzed after 48 h by SDS-PAGE and immunoblotting. Recombinant proteins with the expected sizes were detected with an anti-mCherry (A) or an anti-FLAG antibody (B). The endogenous protein actin was used as a loading control.
(TIF)

Figure S14 The SA4645 antibody specifically recognizes YFP-tagged ATXN1 fusion proteins in crude cell extracts. COS-1 cells were transiently transfected with plasmids encoding YFP-tagged ATXN1Q30, ATXN1Q82, ATXN1Q30^NT^, ATXN1Q82^NT^, ATXN3Q25 or ATXN3Q73 fusion proteins and cell extracts were analyzed by SDS-PAGE and immunoblotting using the anti-ATXN1 antibody SA4645. We observed that all YFP-tagged ATXN1 and ATXN3 fusion proteins were detected with an anti-GFP antibody (A). In contrast, the SA4645 antibody detects ATXN1 but not ATXN3 fusion proteins in cell extracts (B), indicating that the antibody is specific for ATXN1. An anti-actin antibody was utilized to detect endogenous actin in cell extracts (loading control). Abbreviations: IB – immunoblot; a-GFP – anti-GFP antibody; a-ATXN1 - anti-ATXN1 antibody SA4645.
(TIF)

Table S1 List of human genes encoding potential ATXN1 toxicity and aggregation modifiers. Genes encoding putative modifier proteins were predicted using the available literature information from modulator studies in lower model organisms. In addition, interaction partners of polyQ-containing disease proteins (ATXN1, ATXN2, ATXN3 and ATXN7) were selected as potential modulator proteins.
(XLS)

Table S2 Human genes encoding YFP-ATXN1Q82^NT^ toxicity or aggregation modulators. The genes encoding YFP-ATXN1Q82^NT^ toxicity modulators were initially identified in cDNA overexpression experiments in COS-1 cells. In further studies their effects on YFP-ATXN1Q82^NT^ aggregation was investigated. Finally, we examined whether identified YFP-ATXN1Q82^NT^ toxicity modulators also influence YFP-ATXN3Q73 toxicity and aggregation in COS-1 cells.
(XLS)

Table S3 Selected genes for siRNA knock-down experiments in cell-based assays. 15 genes encoding potential ATXN1Q82^NT^ toxicity modifiers were investigated in cell-based siRNA knock-down experiments.
(XLS)

Table S4 Literature-based annotation of potential cellular functions of ATXN1Q82^NT^ toxicity modifiers. ATXN1Q82^NT^ toxicity modifiers were categorized using the available literature information retrieved from PubMed.
(XLS)

Table S5 Predicting coiled-coil domains in ATXN1Q82^NT^ toxicity modifiers. Coiled-coil (CC) domains in modifier proteins were predicted using the COILS program and a window size of 28 amino acids.
(XLS)

Table S6 Oligonucleotides for PCR amplification of cDNA fragments. PCR amplified cDNA fragments were utilized for constructing Gateway compatible entry plasmids. We generated cDNA fragments for the proteins MED15CC, MED15ΔCC, ATXN3Q25, ATXN3Q73, ATXN1Q30, ATXN1Q82, ATXN1Q30^NT^ and ATXN1Q82^NT^.
(XLS)

Table S7 List of cDNA entry clones for construction of destination plasmids.
(XLS)

Table S8 List of destination plasmids for expression of cDNAs in *E. coli* and mammalian cells.
(XLS)

Table S9 List of oligonucleotides for qPCR experiments.
(XLS)
