# Supplementary figures and images for: Identification of Human Proteins That Modify Misfolding and Proteotoxicity of Pathogenic Ataxin-1
Source: PLoS Genet. 2012 Aug 16;8(8):e1002897. doi: 10.1371/journal.pgen.1002897 (PMC3420947; doi:10.1371/journal.pgen.1002897)

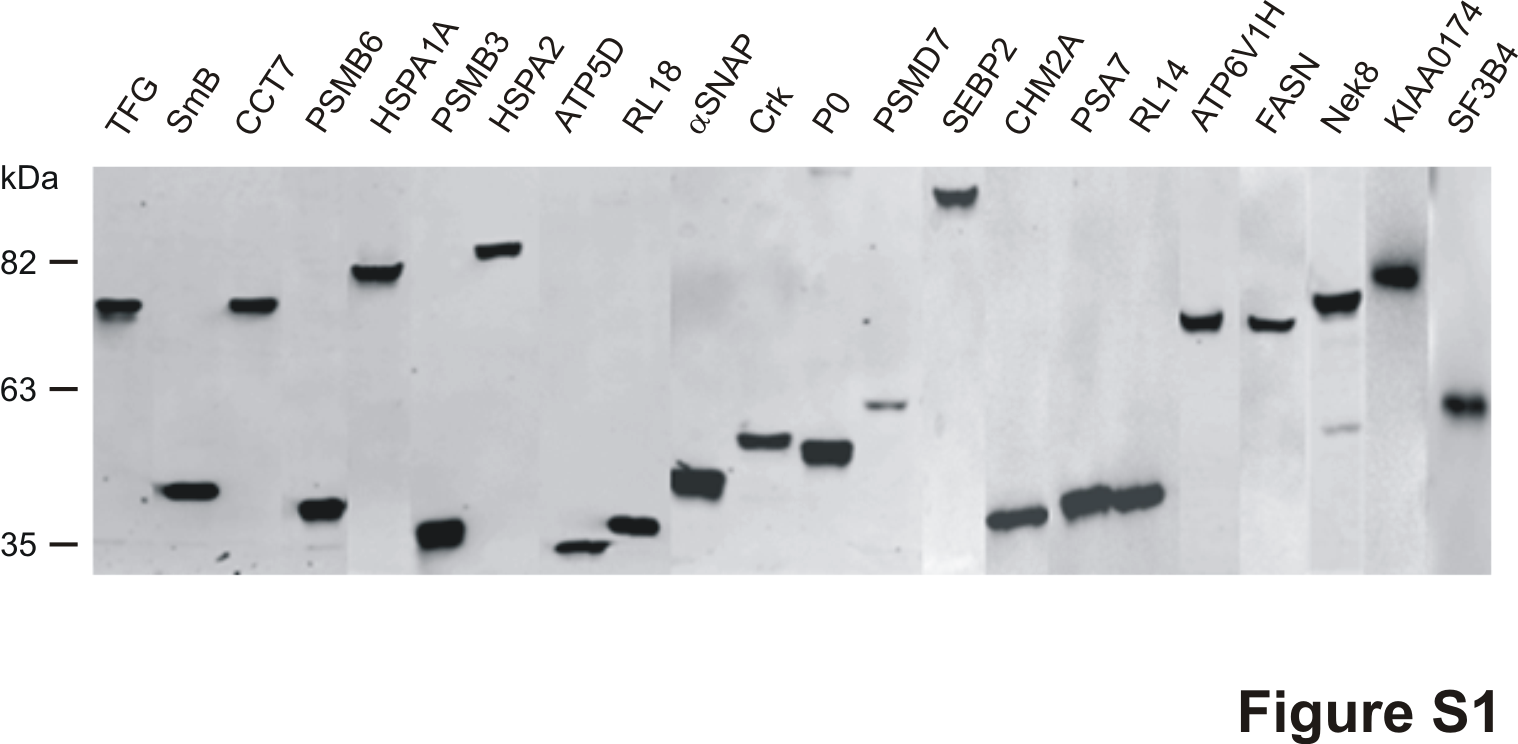

Supplement: Figure S1 — Western blot analysis of FLAG-tagged modulator proteins. Plasmids encoding FLAG-tagged modifier proteins were randomly selected and transfected into COS-1 cells. Protein extracts were prepared after 48 h and analyzed by SDS-PAGE and immunoblotting using the anti-FLAG antibody M2. The same amount of total protein was loaded in each lane. (TIF) [file pgen.1002897.s001.tif]

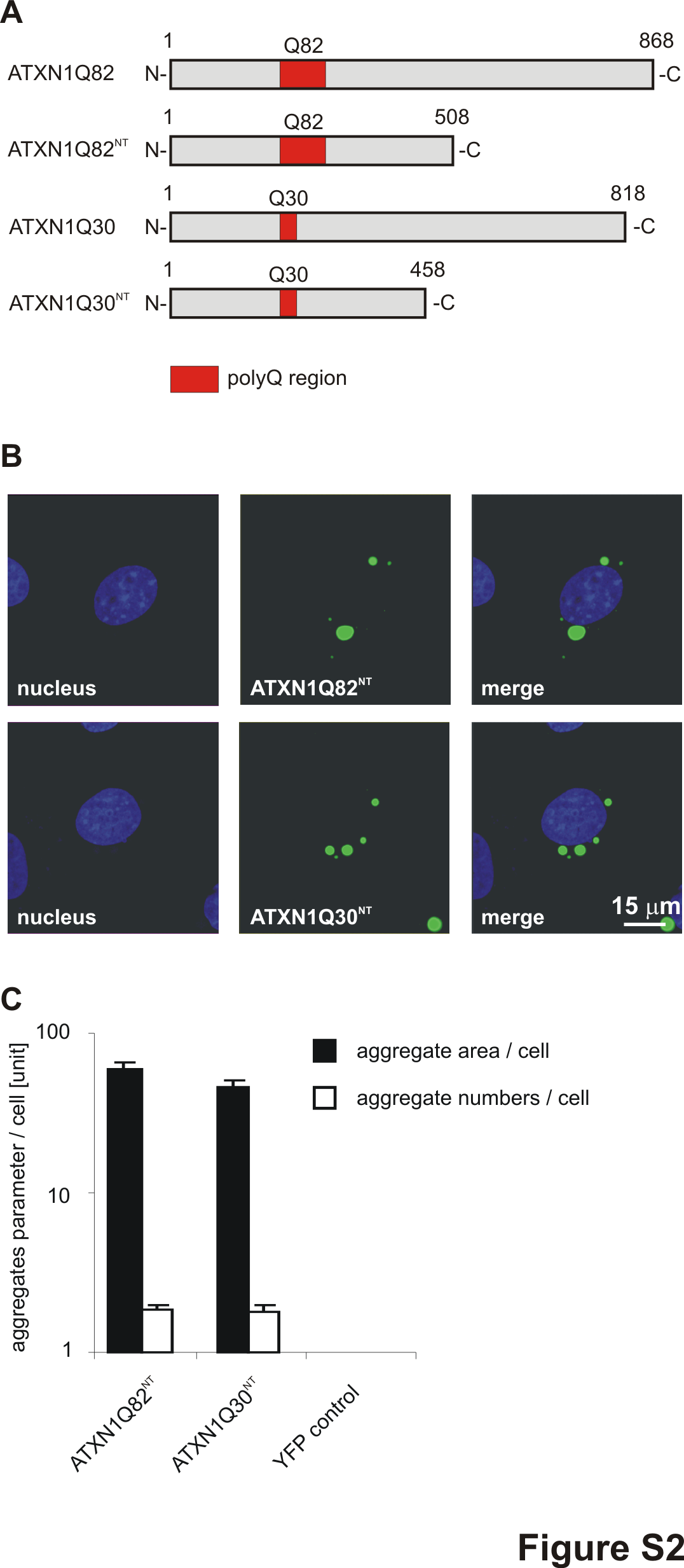

Supplement: Figure S2 — Detection and quantification of YFP-ATXN1Q30NT or YFP-ATXN1Q82NT aggregates in COS-1 cells. (A) A schematic representation of full-length and truncated ATXN1 proteins with pathogenic and non-pathogenic polyQ tracts. (B) Confocal microscopy pictures of YFP-ATXN1Q82NT and YFP-ATXN1Q30NT protein aggregates detected in COS-1 cells. Cells were analyzed after 48 h. Nuclei are stained with Hoechst 33342 (blue) and YFP-ATXN1Q82NT and YFP-ATXN1Q30NT protein deposits are shown in green. (C) Quantification of YFP-ATXN1Q30NT and YFP-ATXN1Q82NT aggregates in COS-1 cells using a high-content screening cell analysis system (Arrayscan VTI, Thermo Scientific). (TIF) [file pgen.1002897.s002.tif]

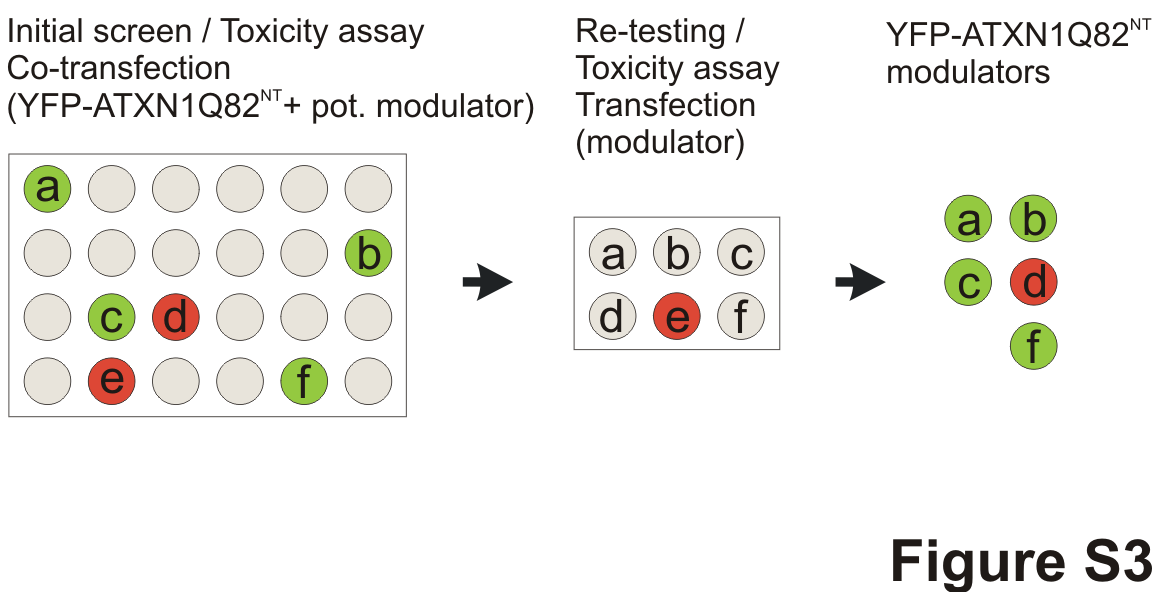

Supplement: Figure S3 — Schematic overview of modifier cytotoxicity screening strategy. COS-1 cells were co-transfected with pairs of plasmids encoding YFP-ATXN1Q82NT and a potential toxicity modifier. Human proteins that either enhance (green) or suppress (red) the toxicity of pathogenic YFP-ATXN1Q82NT are identified in a cell-based screen and subsequently retested in cells in the absence YFP-ATXN1Q82NT fusion protein. Modulators were only considered for further investigations when they specifically influence YFP-ATXN1Q82NT cytotoxicity in cell-based assays. (TIF) [file pgen.1002897.s003.tif]

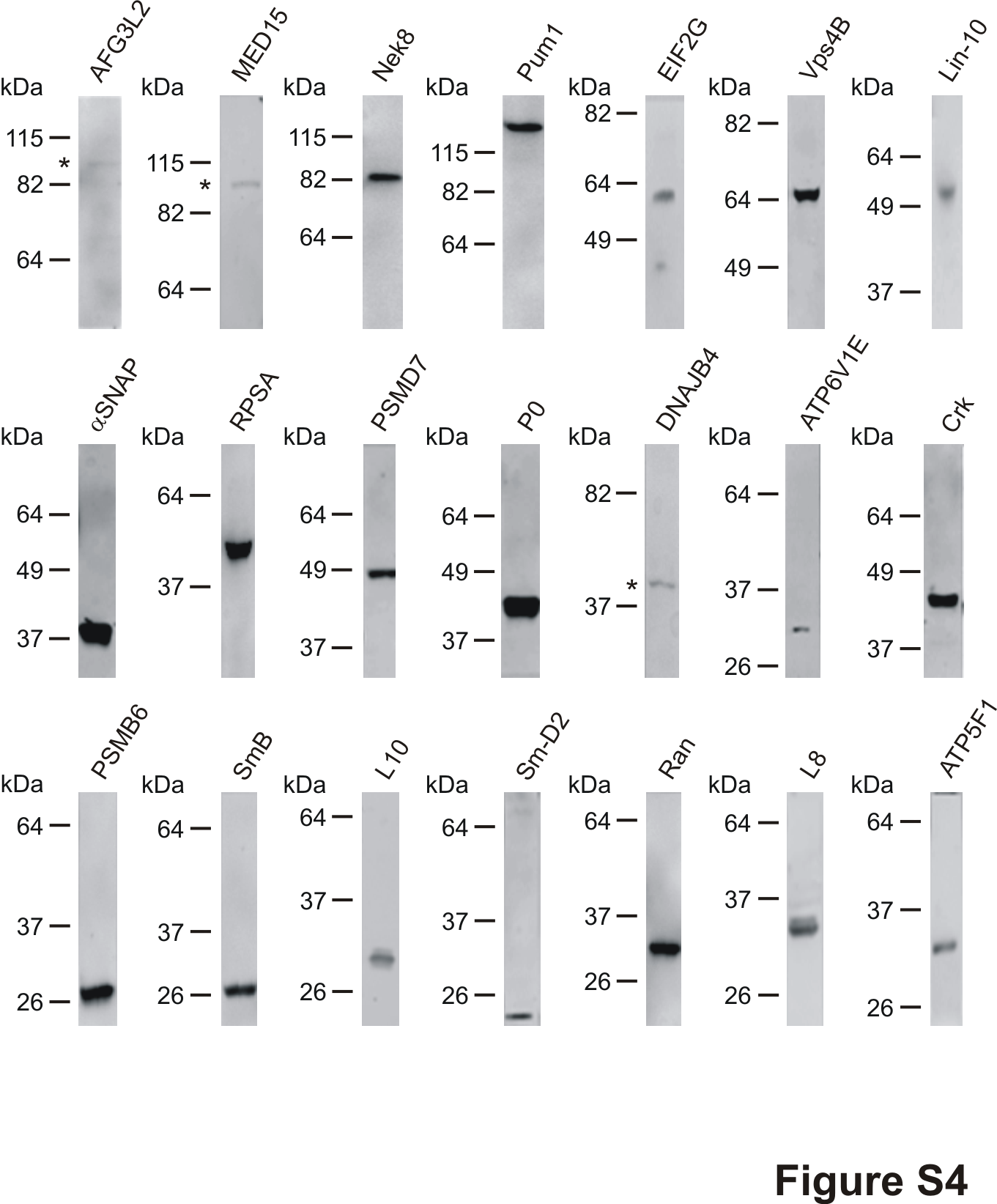

Supplement: Figure S4 — Western blot analysis confirms the production of YFP-ATXNQ82NT toxicity modifiers in COS-1 cells. Overproduction of ATXN1Q82NT cytotoxicity modifiers in transiently transfected COS-1 cells was analyzed after 48 h by SDS-PAGE and immunoblotting using the anti-FLAG antibody M2. Asterisks indicate modifier proteins. (TIF) [file pgen.1002897.s004.tif]

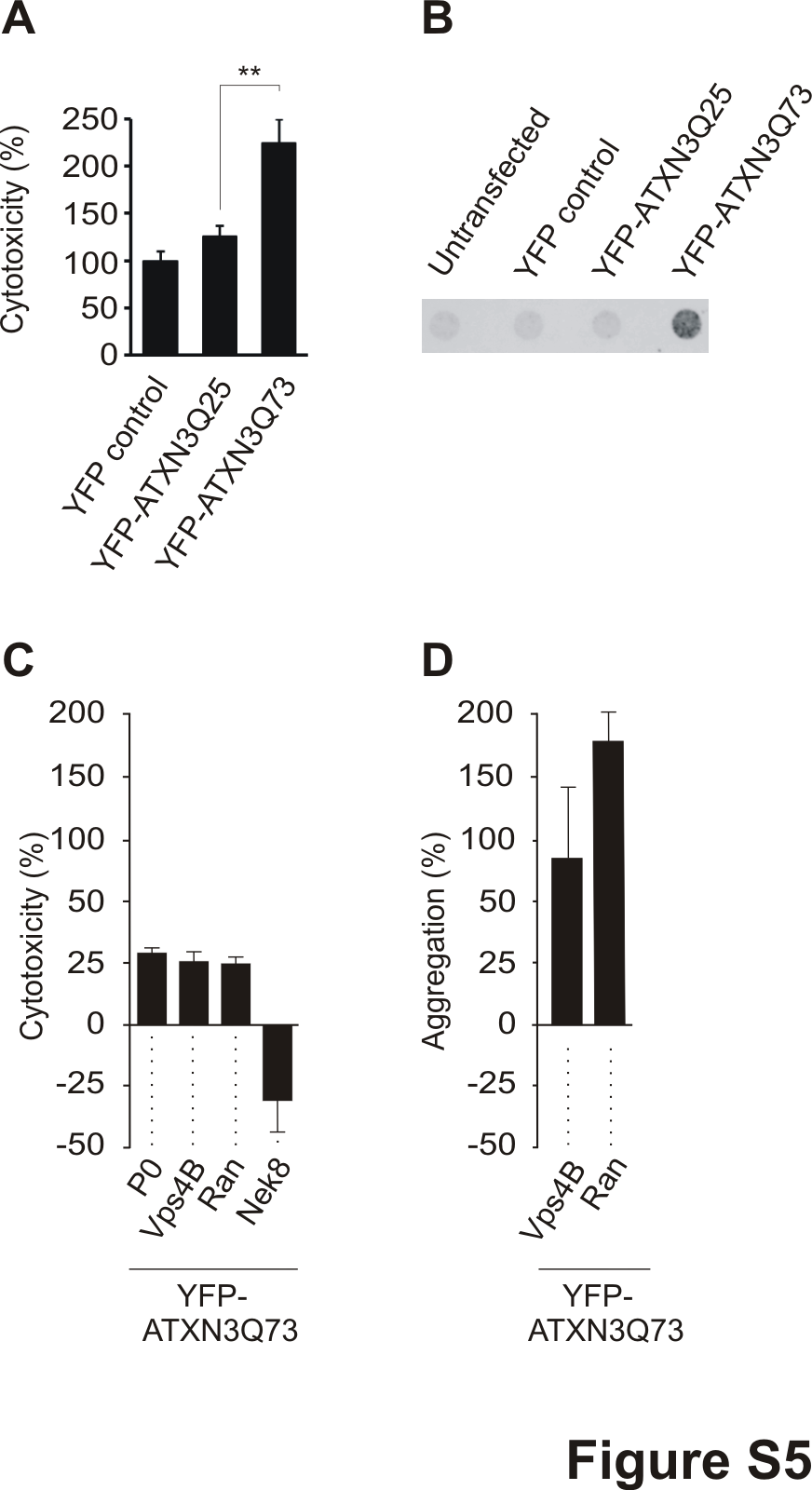

Supplement: Figure S5 — Effects of human proteins on pathogenic ATXN3 cytotoxicity and aggregation. (A) Relative caspase 3/7 activity change induced by overproduction of proteins YFP-ATXN3Q25 or YFP-ATXN3Q73 in COS-1 cells compared to YFP overproducing cells. Caspase 3/7 activity was significantly increased in cells producing the ATXN3 fusion protein with a pathogenic polyQ tract compared to the wild-type protein (Student's t-test, ** p<0.01, n = 3). Error bars indicate SD. (B) Detection of SDS insoluble protein aggregates by filter retardation assay. The YFP-ATXN3Q73 fusion protein forms SDS-insoluble protein aggregates. Equal amounts of total protein were loaded. (C) Effects of modifier proteins on YFP-ATXN3Q73 cytotoxicity; 21 previously identified YFP-ATXN1Q82NT toxicity modifiers (Figure 1E) were systematically tested in cell-based assays. We found that only the proteins P0 (RPLP0), Vps4B (VPS4B), Ran (RAN) and Nek8 (NEK8) influence YFP-ATXN3Q73 toxicity. (D) Effects of modifier proteins on YFP-ATXN3Q73 aggregation; overproduction of Ran and Vps4B enhanced YFP-ATXN3Q73 aggregation. (TIF) [file pgen.1002897.s005.tif]

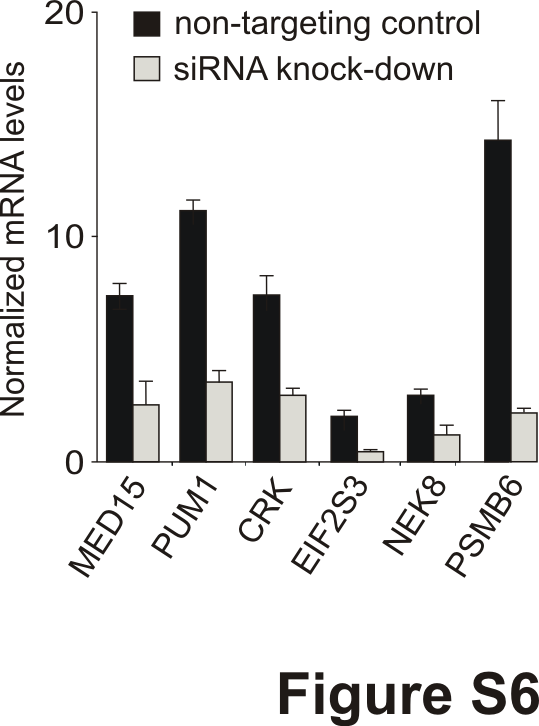

Supplement: Figure S6 — siRNA treatment reduces the expression of modifier genes in mammalian cells. siRNA treated COS-1 cells were analyzed by qRT-PCR. siRNA knock-down of six randomly selected target genes caused a reduction of mRNA levels by 50–90% in comparison to the non-targeting control. mRNA levels were normalized to the mRNA levels of β-actin. Data is shown as mean ± SD for three independent experiments performed in triplicates. (TIF) [file pgen.1002897.s006.tif]

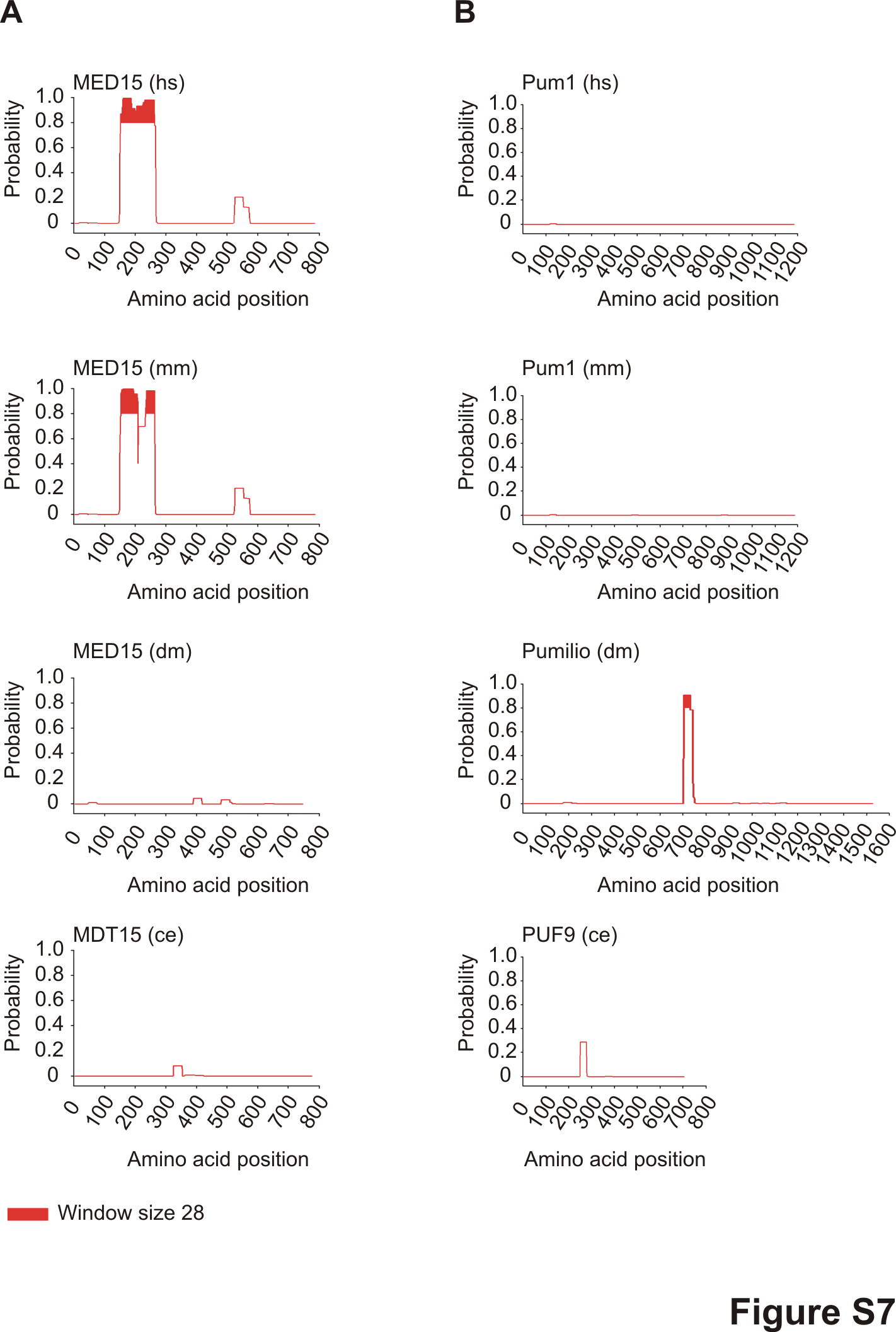

Supplement: Figure S7 — Prediction of coiled-coil domains in related MED15 and Pum1 proteins. Coiled-coil domains in proteins were predicted using the COILS program with a window size of 28 amino acids. (A) Predicted CC domain of human MED15 and its orthologues. (B) Predicted CC domain of human Pum1 and its orthologues. Abbreviations: hs, Homo sapiens; mm, Mus musculus; dm, Drosophila melanogaster; ce, Caenorhabditis elegans. (TIF) [file pgen.1002897.s007.tif]

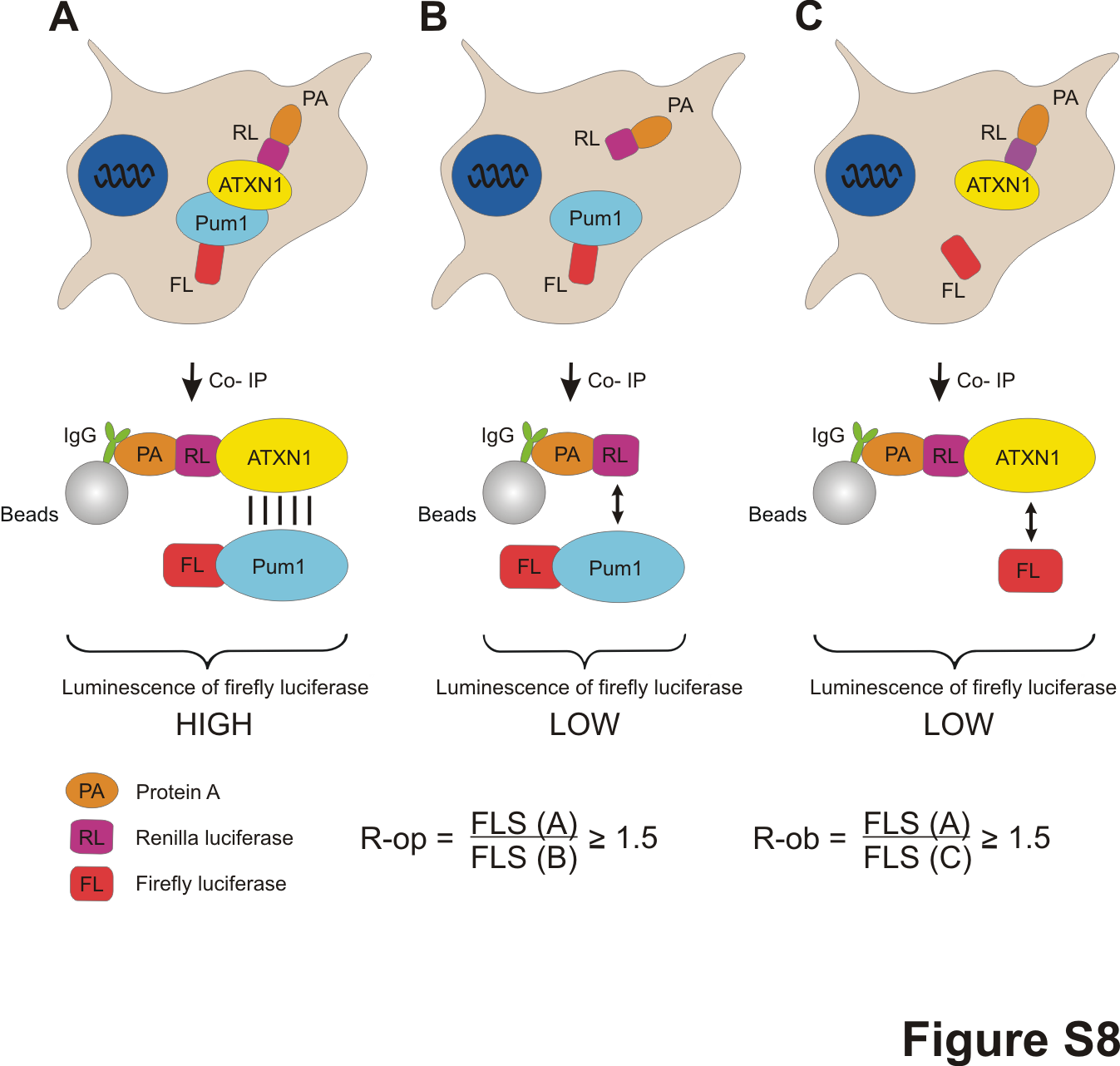

Supplement: Figure S8 — Schematic representation of a cell-based LUMIER co-immunoprecipitation assay. To investigate the interaction between the proteins ATXN1Q30 (bait) and Pum1 (prey) the fusion protein pairs (A) PA-RL-ATXN1Q30/FL-Pum1, (B) PA-RL/FL-Pum1 and (C) PA-RL-ATXN1Q30/FL were co-produced in HEK293 cells. After 48 h the cells were lysed and protein extracts were applied to IgG coated magnetic beads (Dynabeads; Invitrogen) for co-immunoprecipitation (co-IP) of interacting proteins. Following the co-IP the firefly luminescence signal was determined for all three samples (A–C) using a luminescence plate reader. In sample A, as a result of the interaction between PA-RL-ATXN1Q30 and FL-Pum1 a high luminescence signal was detected. In comparison, relatively low signals were obtained in control samples B and C. Using the measured values we calculated the binding ratios R-op and R-ob, which are measure for the specificity of the ATXN1Q30 and Pum1 interaction. Based on previous empirical studies with well-characterized interaction test pairs, we define an interaction as positive when the calculated R-op and R-ob ratios are >1.5. (TIF) [file pgen.1002897.s008.tif]

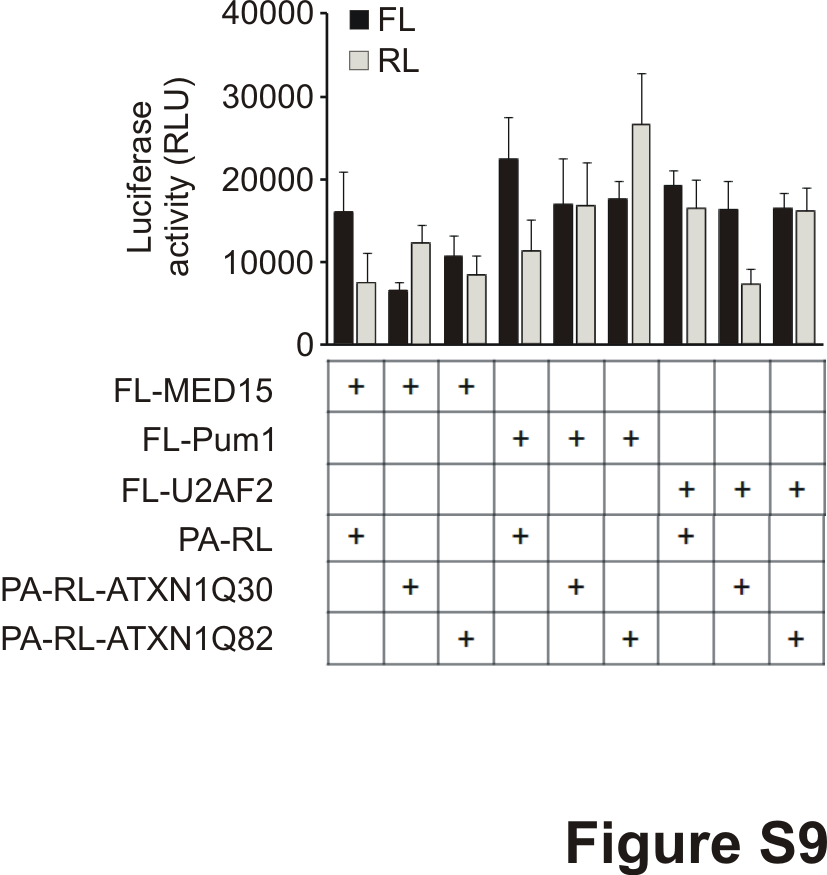

Supplement: Figure S9 — Detection of firefly luciferase and Renilla luciferase tagged fusion proteins in HEK293 cell extracts. Production of Renilla and firefly luciferase-tagged fusion proteins in HEK293 cells was monitored by quantification of luminescence activity of protein extracts. Data are shown as mean ± SD for three separate experiments performed in triplicates. Abbreviations: FL - firefly luciferase; RL - Renilla luciferase; PA - protein A; RLU - relative light units. (TIF) [file pgen.1002897.s009.tif]

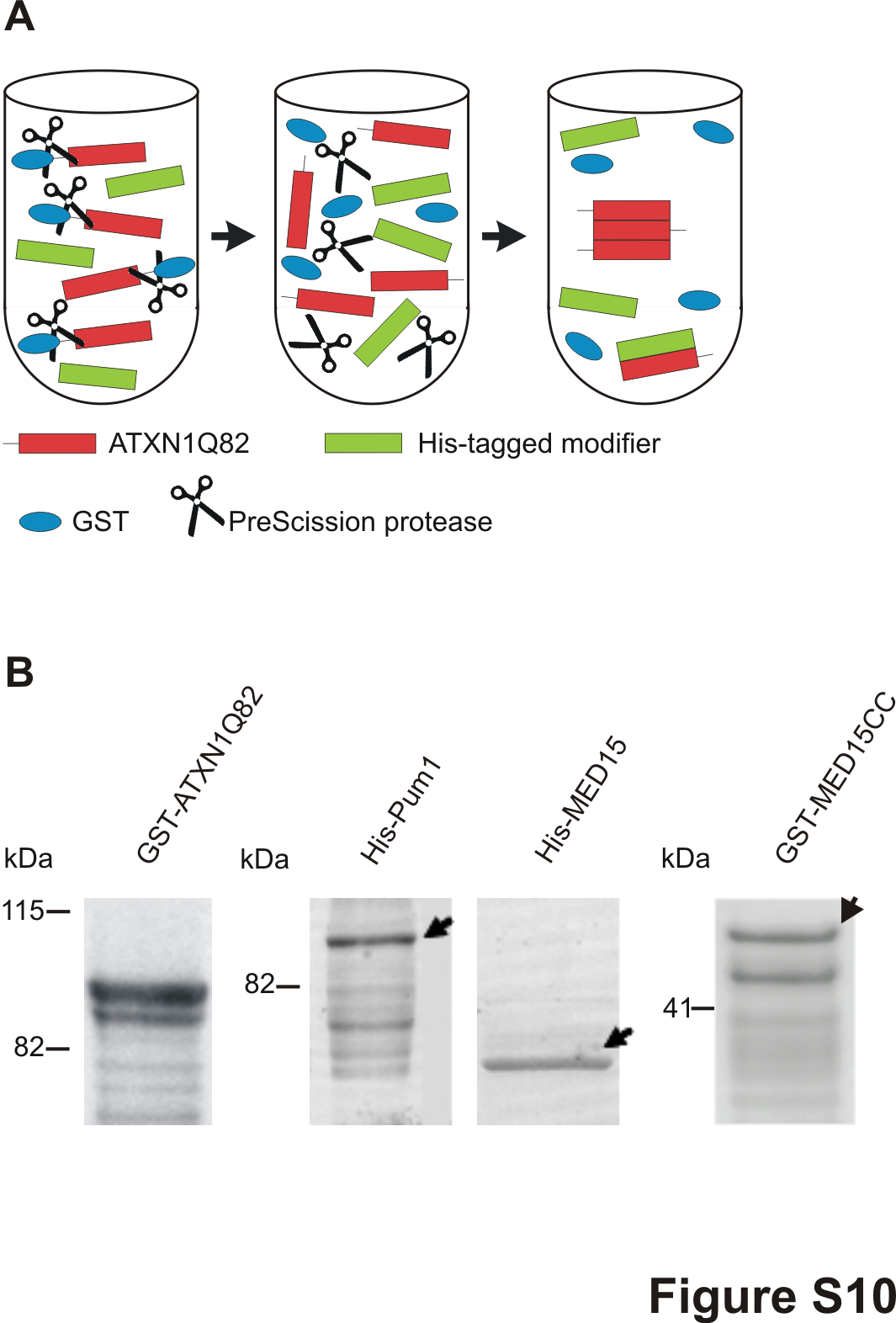

Supplement: Figure S10 — Purification of recombinant GST- and His-tagged fusion proteins for cell-free aggregation assays. (A) Schematic representation of a spontaneous ATXN1Q82 aggregation reaction in the presence of a modifier protein. Aggregation of ATXN1Q82 is initiated by proteolytic cleavage of the fusion protein GST-ATXN1Q82 with PreScission protease. Insoluble ATXN1Q82 aggregates form spontaneously over time and are quantified by filter retardation assay. The effects of modifiers can be assessed in a time and concentration dependent manner. (B) GST- and His-tagged fusion proteins were produced in E. coli and purified by affinity chromatography. Analysis by SDS-PAGE and Coomassie staining confirmed that recombinant fusion proteins with expected sizes are produced in E. coli. (TIF) [file pgen.1002897.s010.tif]

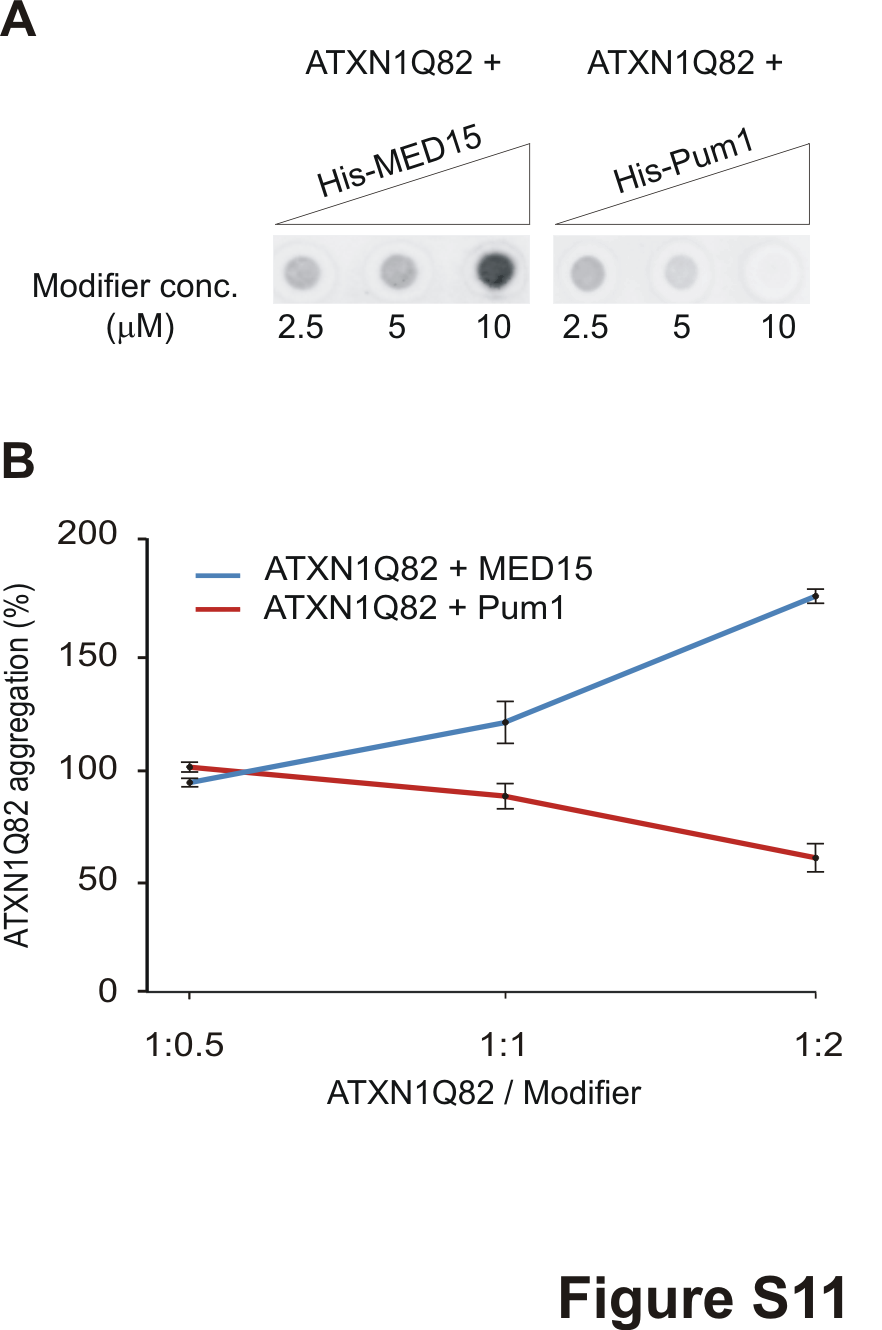

Supplement: Figure S11 — The recombinant proteins His-MED15 and His-Pum1 influence spontaneous ATXN1Q82 aggregation in a concentration-dependent manner. (A) Detection of SDS-insoluble ATXN1Q82 aggregates by filter retardation assay. GST-ATXN1Q82 (5 µM) was incubated with PreScission protease and modulator proteins (2.5, 5 or 10 µM) and formation of insoluble ATXN1Q82 protein aggregates was quantified after 48 h by filter assay. His-MED15 in a concentration dependent manner increased spontaneous ATXN1Q82 aggregation, while His-Pum1 has the opposite effect. SDS-insoluble ATXN1Q82 protein aggregates retained on filter membranes were detected using the anti-ATXN1 antibody SA4645. (B) Quantification of filter retardation assay data with the AIDA densitometry software. Error bars represent SD of three independent experiments. (TIF) [file pgen.1002897.s011.tif]

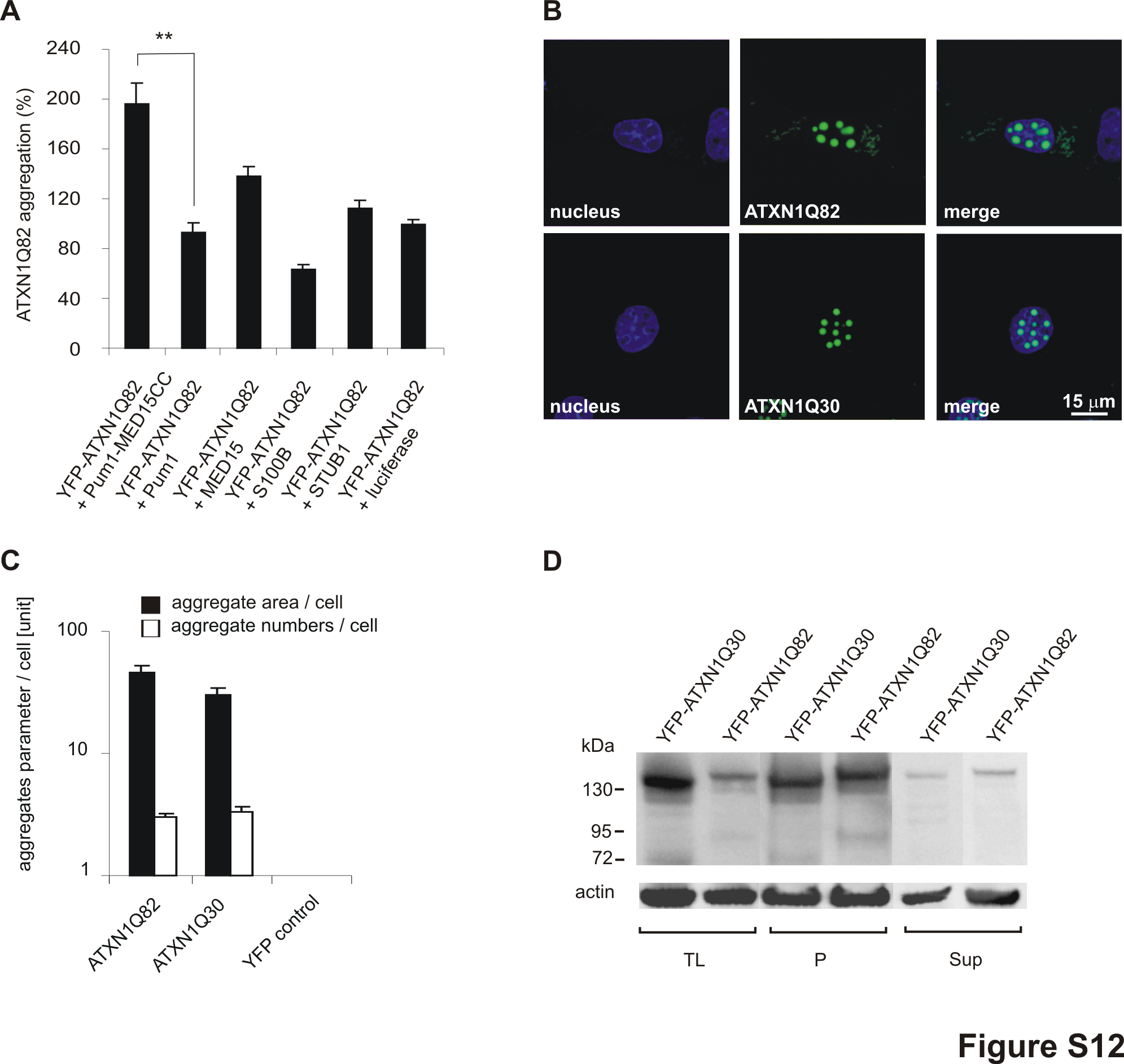

Supplement: Figure S12 — Effects of modulator proteins on aggregation of full-length YFP- ATXN1Q82. (A) Effects of mCherry-tagged proteins Pum1-MED15CC, Pum1, MED15, S100B, STUB1 and luciferase on spontaneous YFP-ATXN1Q82 aggregation in human neuroblastoma SH-EP cells. The formation of polyQ-containing ATXN1 aggregates was quantified by fluorescence imaging after 48 h. We found that the hybrid protein Pum1-MED15CC in comparison to the wild-type Pum1 and the luciferase control protein (100%) readily promotes YFP-ATXN1Q82 aggregation, confirming the results obtained with the YFP-ATXN1Q82NT protein (Figure 6B). An increase of YFP-ATXN1Q82 aggregates was also observed in MED15 and STUB1 overproducing SH-EP cells. In strong contrast, a suppression of spontaneous YFP-ATXN1Q82 aggregation was detected with the control protein S100B. Data are shown as mean ± SD from three independent experiments. Experiments were performed in triplicates. Student's t-test was used for statistical comparisons, p<0.05. (B) Analysis of spontaneous YFP-ATXN1Q30 and YFP-ATXN1Q82 aggregation in COS-1 cells by confocal microscopy. COS-1 cells were transfected with plasmids encoding the full-length ATXN1 proteins YFP-ATXN1Q30 and YFP-ATXN1Q82 and after 48 h cells were investigated by confocal microscopy. Nuclei were stained with Hoechst (blue). We found that both wild-type and mutant ATXN1 fusion proteins form nuclear inclusions in COS-1 cells (green). (C) Quantification of aggregate number and size in YFP-ATXN1Q30 and YFP-ATXN1Q82 overproducing cells a high-content screening cell analysis system (Arrayscan VTI, Thermo Scientific). (D) Detection of YFP-ATXN1Q30 and YFP-ATXN1Q82 fusion proteins in COS-1 cell extracts by SDS-PAGE and immunoblotting. Proteins were detected using anti-GFP and anti-actin antibodies. Abbreviations: TL - total lysate; P – pellet; Sup – supernatant. (TIF) [file pgen.1002897.s012.tif]

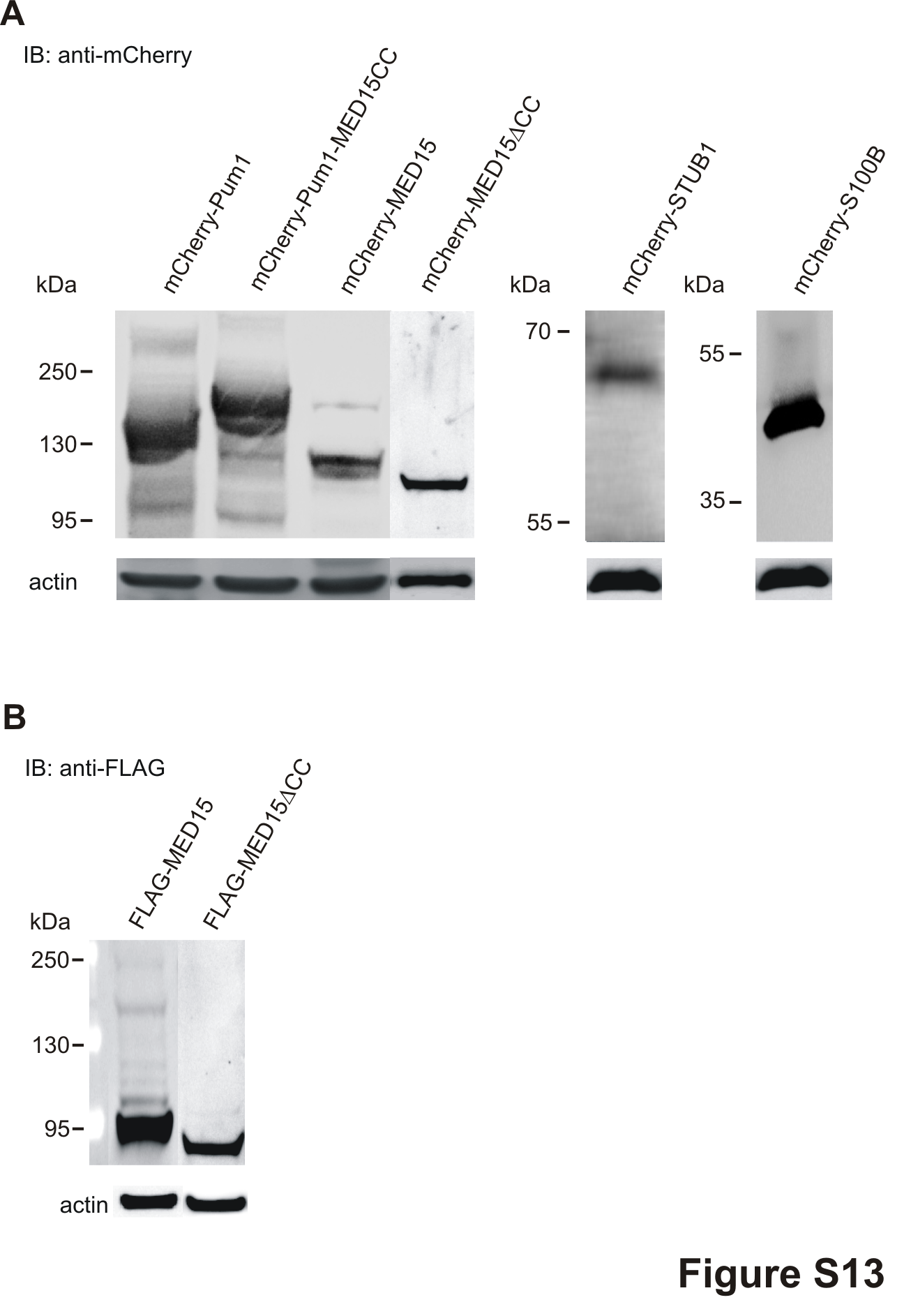

Supplement: Figure S13 — Production of mCherry- and FLAG-tagged modifier proteins in mammalian cells. SH-EP neuroblastoma cells were transfected with plasmids encoding (A) mCherry- or (B) FLAG-tagged modifier proteins; protein extracts were analyzed after 48 h by SDS-PAGE and immunoblotting. Recombinant proteins with the expected sizes were detected with an anti-mCherry (A) or an anti-FLAG antibody (B). The endogenous protein actin was used as a loading control. (TIF) [file pgen.1002897.s013.tif]

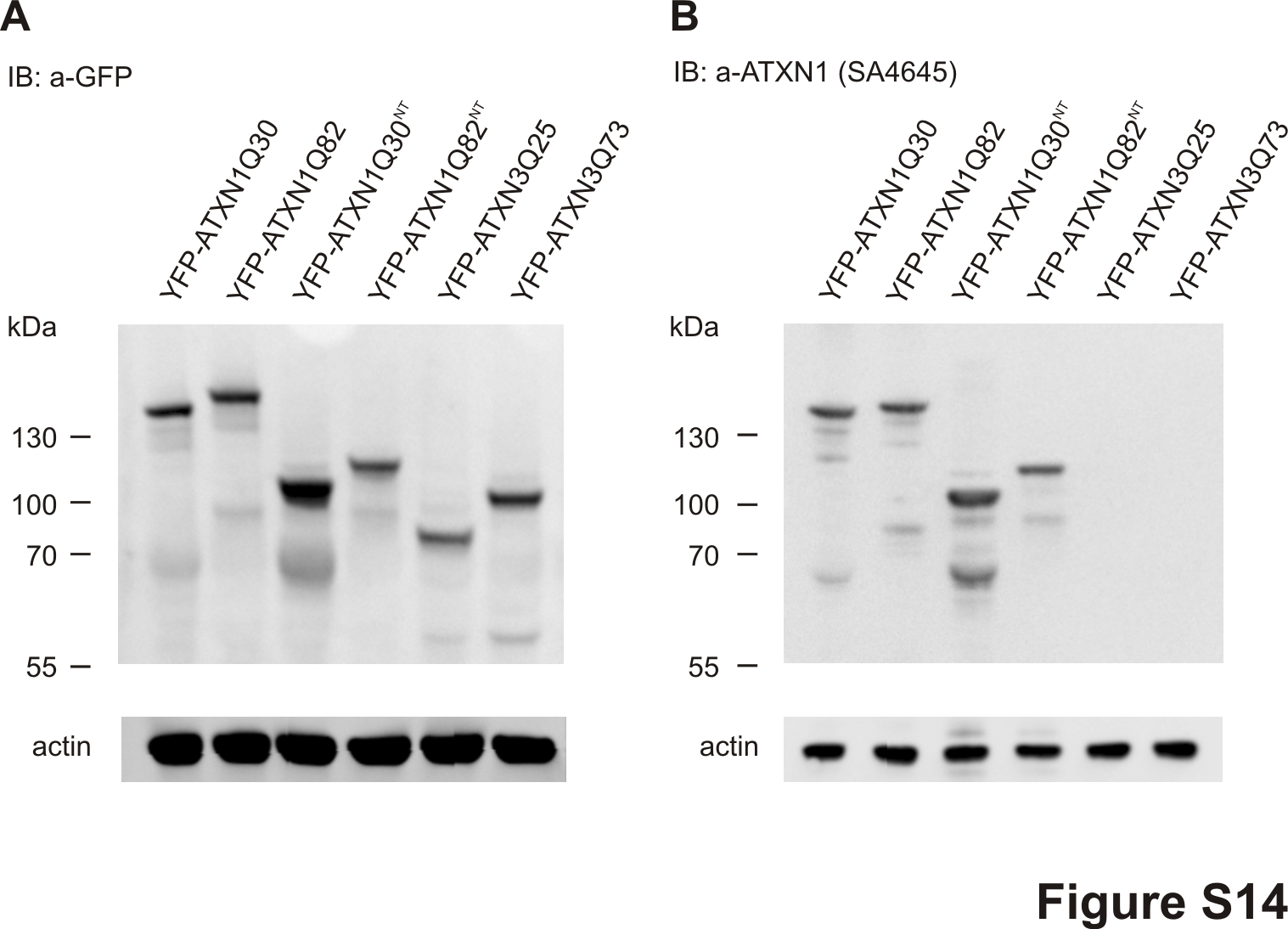

Supplement: Figure S14 — The SA4645 antibody specifically recognizes YFP-tagged ATXN1 fusion proteins in crude cell extracts. COS-1 cells were transiently transfected with plasmids encoding YFP-tagged ATXN1Q30, ATXN1Q82, ATXN1Q30NT, ATXN1Q82NT, ATXN3Q25 or ATXN3Q73 fusion proteins and cell extracts were analyzed by SDS-PAGE and immunoblotting using the anti-ATXN1 antibody SA4645. We observed that all YFP-tagged ATXN1 and ATXN3 fusion proteins were detected with an anti-GFP antibody (A). In contrast, the SA4645 antibody detects ATXN1 but not ATXN3 fusion proteins in cell extracts (B), indicating that the antibody is specific for ATXN1. An anti-actin antibody was utilized to detect endogenous actin in cell extracts (loading control). Abbreviations: IB – immunoblot; a-GFP – anti-GFP antibody; a-ATXN1 - anti-ATXN1 antibody SA4645. (TIF) [file pgen.1002897.s014.tif]
